# Supramolecular Metallacycles and Their Binding of Fullerenes

**DOI:** 10.1002/chem.201905390

**Published:** 2020-03-05

**Authors:** Christian R. Göb, Andreas Ehnbom, Lisa Sturm, Yoshito Tobe, Iris M. Oppel

**Affiliations:** ^1^ Institute of Inorganic Chemistry RWTH Aachen University Landoltweg 1 52074 Aachen Germany; ^2^ Department of Chemistry Texas A&M University P.O. Box 30012 College Station TX 77843-3012 USA; ^3^ Division of Frontier Materials Science Graduate School of Engineering Science Osaka University Toyonaka Osaka 560-8531 Japan

**Keywords:** cage compounds, density functional calculations, fullerenes, host–guest systems, self-assembly

## Abstract

The synthesis of a new triaminoguanidinium‐based ligand with three *tris*‐chelating [NNO]‐binding pockets and *C*_3_ symmetry is described. The reaction of *tris*‐(2‐pyridinylene‐*N*‐oxide)triaminoguanidinium salts with zinc(II) formate leads to the formation of cyclic supramolecular coordination compounds which in solution bind fullerenes in their spherical cavities. The rapid encapsulation of C_60_ can be observed by NMR spectroscopy and single‐crystal X‐ray diffraction and is verified using computation.

## Graphical abstract

**Cage bound**: Synthesis of a new triaminoguanidinium‐based ligand with three *tris*‐chelating [NNO] binding pockets and *C*_3_‐symmetry is described. Reaction with zinc(II) formate leads to the formation of cyclic supramolecular coordination compounds that in solution are able to bind fullerenes in their spherical cavities. The rapid encapsulation of C_60_ is observed by NMR spectroscopy and X‐ray diffraction and is validated by DFT.

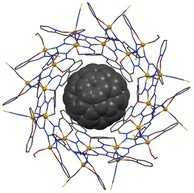

## Introduction

**Figure 1:**
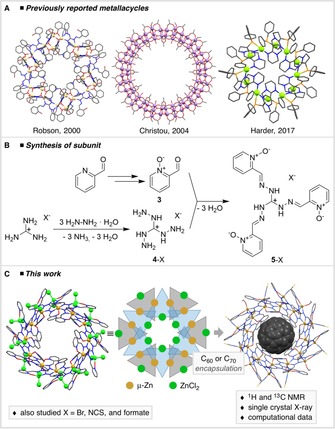
(a) Previously reported supramolecular metallacycles. Hydrogen atoms and solvent molecules were omitted for clarity. Different scales were applied and metal ions highlighted as spheres. (b) Synthesis of the ligands [H_3_(pyO)_3_L]X (**5**‐X), (X=Cl^−^, NCS^−^, BF_4_^−^). (c) Supramolecular metallacycle [Zn_24_Cl_24_{(pyO)_3_L}_12_] (ZnCl_2_ in green), schematic drawing and inclusion complex C_60_⊂**9**.

The development of supramolecular coordination compounds and their corresponding cages has attracted wide interest in recent years.^1^ They have the potential to transport chemicals from one location to another in a specific manner, for example, being used in drug delivery or as contrast agents.^2^ The structural design of these containers requires precise and complementary building blocks but they are not limited to the incorporation of small organic molecules.^3^ There are only a few examples of toroid coordination compounds and larger aggregates in the literature (Figure 1 A).^4^ Those compounds can be used as single molecular magnets or in the separation of fullerenes.^5^ Stang introduced the concept of using building blocks to form cage compounds,^6^ whereas the groups of Saalfrank, Fujita, and Nitschke used cages to stabilize reactive species like white phosphorous P_4_ and organometallic complexes.6b, 7

Toroids can be useful in binding guest molecules. Covalently bonded systems are quite common, for example, cyclodextrins, cucurbiturils and cryptands which bind cations,^8^ anions,^9^ or hydrophobic molecules^10^ by variation of their peripheral decoration. Toroidal coordination cages are also able to bind guest molecules and separate fullerenes.^11^

These systems are challenging to model using density functional theory (DFT) due to their large size. Herein we demonstrate not only that a geometry‐optimized structure of a large empty metallacycle can be obtained, but also the C_60_ and C_70_ encapsulated supramolecular entity. We were able to compute spectroscopic properties and assign the C_60_ signals in both the free state and bound inside of the metallacycle (Figure 1 C). There are a large number of studies reporting the computations of C_60_ itself. However, publications focusing on the interaction of C_60_ with other macrocycles are limited. Most studies including these interactions involve smaller, and often purely organic macrocyclic systems.^12^ To the best of our knowledge, this is the first study to computationally investigate such large interacting systems (>4000 electrons) using non‐truncated models.

## Results and Discussion

**Figure 2:**
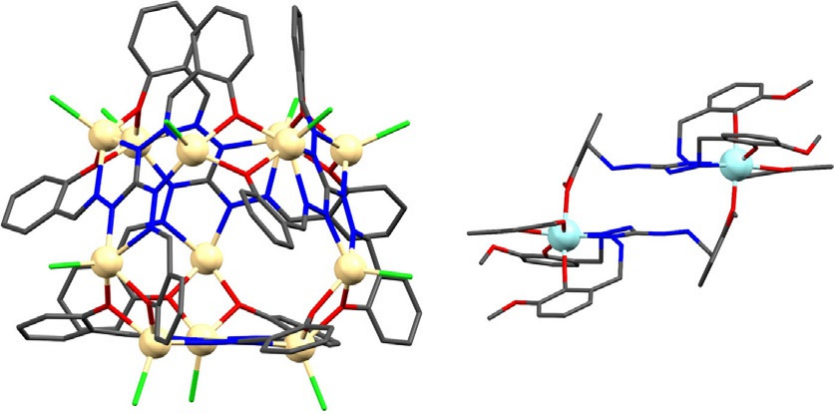
M_12_L_4_ tetrahedron and M_2_L_2_ dimer (M=Cd, Sn, respectively). Hydrogen atoms and solvent molecules were omitted for clarity.^[14, 13a]^

Our group demonstrated the synthesis of supramolecular structures by self‐assembly of *C*_3_‐symmetric building blocks with three *tris*‐chelating binding pockets and suitable co‐ligands (analogous to Figure 1 B).^13^ Counter ions or solvent molecules typically serve as templates in the synthesis, so that discrete coordination cages like M_12_L_4_ (Figure 2, left), M_18_L_6_, or M_24_L_8_ are accessible.13d We did not observe any activation of small molecules with these assemblies, even though a high number of potential catalytically active metal centers are located proximal to each other.

The overall negative charge of these complexes might be the reason why substrates like lactide are not satisfyingly activated. It was thus necessary to increase the amount of positive charges of the resulting coordination compounds by using stronger Lewis acids, such as Sn^IV^ or Zr^IV^, instead of Cd^II^ or Pd^II^.^14^ The resulting complexes typically form dimers or trimers and are able to oligomerize acetone in up to 15 repeating units (Figure 2, right).13d, 15

In this work we address the charge issue by increasing the number of positive charges in the ligand itself, while maintaining the isoelectronic structure of the previously reported ligands. The ligand is prepared from a condensation reaction between 2‐formylpyridine‐*N*‐oxide and the corresponding triaminoguanidinium salts *TAG*‐Cl (**4**‐Cl), *TAG*‐NCS (**4**‐NCS), or *TAG*‐BF_4_ (**4**‐BF_4_), respectively (Figure 1 B).

**Figure 3:**
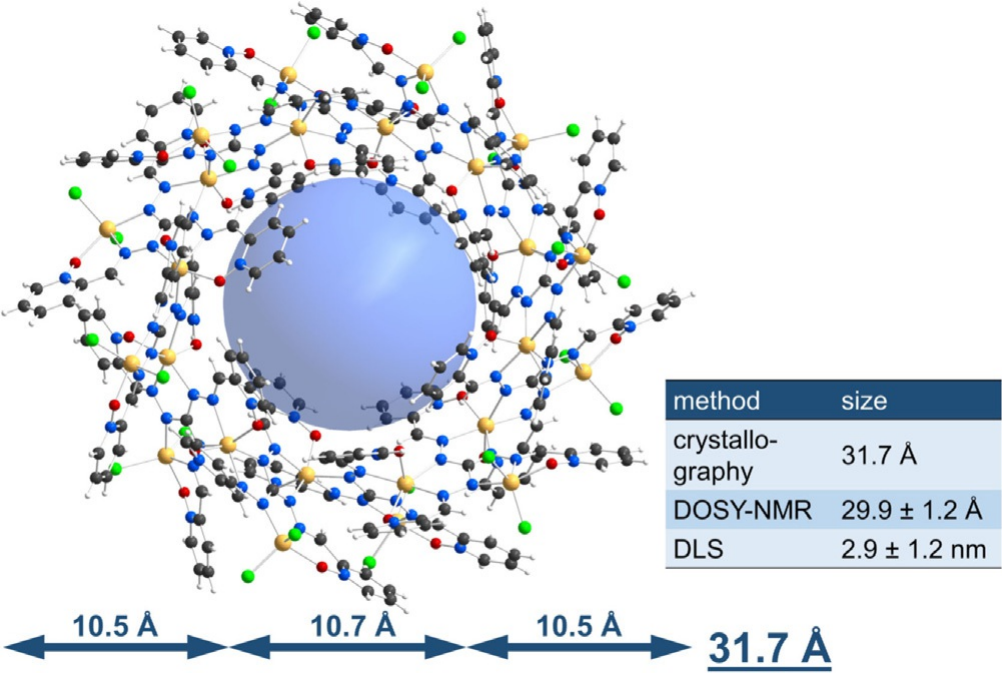
Molecular dimensions of metallacycles by X‐ray diffraction compared to spectroscopic data of **7**. The void space is indicated by the blue sphere.

**Figure 4:**
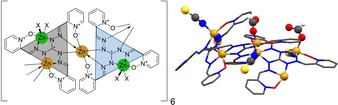
Schematic representation of metallacycles (X=Cl, Br, NCS, O_2_CH) and asymmetric unit of [Zn_24_(NCS)_16_(O_2_CH)_8_{(pyO)_3_L}_12_] **8**. Disordered solvent molecules were removed by the Squeeze routine (Platon) and hydrogen atoms were omitted for clarity.^18^

The synthesis of *N*‐oxide (**3**) requires standard protection and deprotection procedures for the aldehyde moiety. The oxidation of the pyridine nitrogen can be realized under mild conditions by using urea hydrogen peroxide with phthalic anhydride in acetonitrile.^16^ Triaminoguanidinium salts (**4**‐X) are obtained by the amination of guanidinium salts with hydrazine hydrate.^17^ The resulting compounds (**5**‐X) serve as an excellent ligands for Zn^II^. The reaction of [H_3_(pyO)_3_L]Cl (**5**‐Cl) with Zn(O_2_CH)_2_ in *N*,*N*‐dimethylformamide results in the formation of a supramolecular coordination compound [Zn_24_Cl_24_{(pyO)_3_L}_12_] (**6**, Figure 1 C) next to a coordination polymer of unknown composition. This torus‐shaped metallacycle exhibits an outer diameter of 31.7 Å and Zn^II^ ions are octahedrally coordinated between two alternately oriented ligands holding together the assembly (Figure 3 and Figure 4, left). The ZnCl_2_ moieties occupy the remaining [NNO] binding pockets. A spherical cavity of 10.7 Å can be found inside the complex with a pore opening of 8.2 Å. Each value is corrected by the covalent radii of hydrogen or carbon atoms.

An isostructural cyclic coordination oligomer [Zn_24_Br_24_{(pyO)_3_L}_12_] (**7**) can be crystallized from the reaction mixture containing [H_3_(pyO)_3_L]BF_4_, ZnBr_2_ and NaO_2_CH. DOSY‐NMR spectroscopy ([D_6_]DMSO) of **7** shows neither decomposition nor aggregation of the coordination complex in solution. Only one species is detected in addition to solvents and water. The diffusion coefficient of **7** is found to be *D*=(7.36±0.08)×10^7^ cm^2^ s^−1^. The hydrodynamic diameter of **7** can be determined to be 29.9±1.2 Å using the Stokes–Einstein equation, which corresponds well with the observed diameter of the crystal structure (Supporting Information Figure S5 and S6).

**Figure 5:**
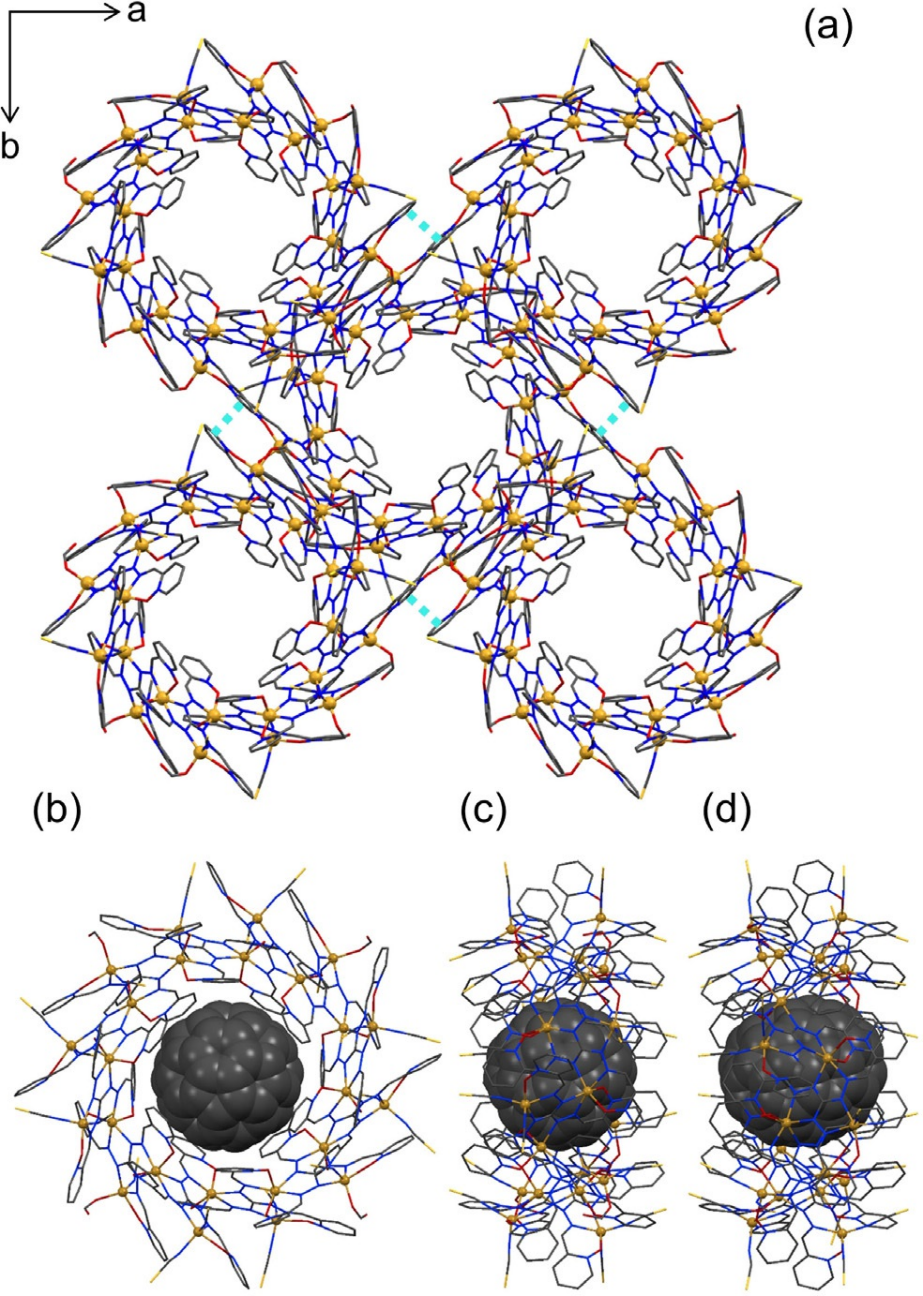
(a) Crystal packing of **8**, π–π‐interactions are highlighted in turquoise. (b,c) C_60_⊂**9**, (d) C_70_⊂**9**. Disordered solvent molecules were removed by the Squeeze routine and hydrogen atoms were omitted for clarity.

Introduction of isothiocyanate leads to the formation of the analogous NCS‐metallacycle [Zn_24_(NCS)_16_(O_2_CH)_8_{(pyO)_3_L}_12_] (**8**) with a 59 % yield. The zinc(II) ions, which were formally occupied by the halides Cl^−^ or Br^−^, share those sites with isothiocyanate and formate co‐ligands (Figure 4, right). The presence of this coordination compound, and the absence of smaller or larger aggregates in the DMSO solution, was confirmed by dynamic light scattering (Supporting Information). Since the co‐ligands point outwards and exhibit a slightly increased steric demand, the system crystallizes in the tetragonal space group *P* $\bar{4}$ 2_1_ *c* with solvent filled channels along the crystallographic *c*‐axis (Figure 5 a). Host‐guest chemistry seems feasible since the cavities of the coordination oligomers should be accessible in the solid state. To our surprise, it was not possible to soak crystals of [Zn_24_(NCS)_16_(O_2_CH)_8_{(pyO)_3_L}_12_] (**8**) with a toluene solution of C_60_, as there was no observed color change.^19^

From these experimental results we decided to encapsulate the fullerenes into the metallacycles in solution. A solution of empty metallacycle (**8**) was added to a toluene solution of C_60_ or C_70_. Single crystals of the respective inclusion compounds were collected after a few days (Figure 5 b–d). The C_60_ and C_70_ are fully incorporated into the cavity of the metallacycle [Zn_24_(NCS)_20_(O_2_CH)_4_{(pyO)_3_L}_12_] (**9**). The lattice parameters underwent a slight change compared to **8**, whereas the space group was maintained. The periphery of the metallacycle was slightly perturbed, presumably due to the change in polarity of the solvent mixture.

**Figure 6:**
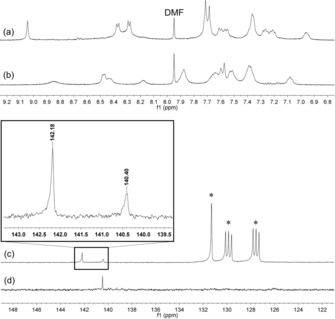
(a,b) ^1^H NMR spectra in [D_6_]DMSO of **8** and C_60_⊂**9** respectively. (c) ^13^C NMR spectrum of **8** (1 equiv) and C_60_ (2 equiv) in [D_6_]DMSO/1,2‐dichlorobenzene‐*d*_4_ 1:1 (v/v). (d) ^13^C NMR spectrum of C_60_⊂**9** in [D_6_]DMSO.

The incorporation of C_60_ in solution is validated by NMR spectroscopy (Figure 6). Crystals of **8** and C_60_⊂**9** were removed from the crystallization solution, washed with THF and dissolved in [D_6_]DMSO. The ^1^H NMR spectrum shows a shift and broadening of the signals due to the molecular tumbling of C_60_. The ^13^C NMR of C_60_⊂**9** shows only one signal for C_60_ which is in agreement with all carbon atoms being chemically and magnetically equivalent. The encapsulated C_60_ can only be detected next to free C_60_ in a highly diluted solvent mixture, and therefore ^13^C‐enriched C_60_ was used. The 2 ppm signal shift from 142.18 ppm (free C_60_) to 140.40 ppm (C_60_⊂**9**) clearly indicates the incorporation of C_60_ in the cavity of the metallacycle.

**Figure 7:**
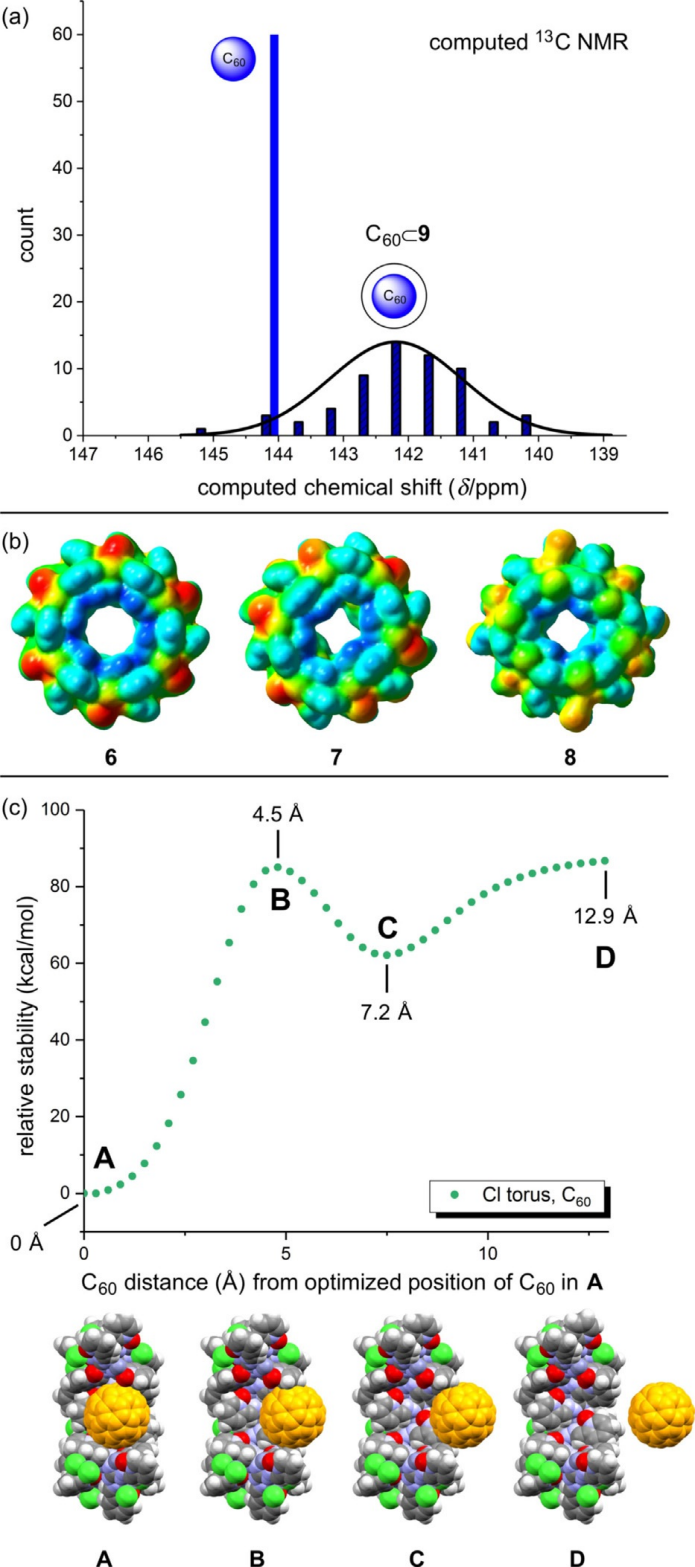
(a) Computed ^13^C NMR of **8** and C_60_⊂**9**, (b) electrostatic potential maps (ESP) of **6**, **7**, and **8**. (c) An electronic energy diagram showing the relative stability (kcal mol^−1^) as a function of the C_60_ distance from its position in the fully optimized state (A) in C_60_⊂**9**. The C_60_ is stepwise extruded.

Calculations predicted the encapsulation of C_60_ by the metallacycle (**6**) to be favorable (Figure 7 c **A**). However, there is an additional local minimum (**C**) where the C_60_ interacts with the periphery of the metallacycle. This has also been observed crystallographically. An analogous binding plot is observed for the C_70_ case (see Supporting Information for details). The use of dispersion corrections in these calculations is critical in order to correctly model the attractive interaction. In addition to the entropy loss, there is an electronic energy barrier (**B**) that C_60_ (and C_70_) must overcome caused by steric interactions between the C_60/70_ and pyridinyl groups located at the entrance of the metallacycle. Due to the nature of these calculations, stepwise single‐point calculations from the fully optimized state (**A**) were applied. The binding energetics are only qualitative. The differences in electrostatic potentials for **6**–**8** are shown in Figure 7 b. The chloride metallacycle has the largest positive inner core while the outside ZnCl_2_ moieties carry the negative potential. This is gradually attenuated in the series of **6**>**7**>**8** with **8** having the least positive inner core. These potentials might be useful in tuning the affinity and specificity for guest molecules.

In agreement with experimental data, spectroscopic assignments using computations (DFT, see Supporting Information for details) predict an approximately 2.0 ppm upfield shift for the encapsulated C_60_⊂**9** species versus the free C_60_ (144.3 ppm vs. 142.2 ppm) (Figure 7 a). The IR signatures of an empty chloride metallacycle (**6**) also match the computed IR spectrum and the fingerprint region contains several characteristic absorptions (Figure S25 in the Supporting Information).

## Summary

We report the synthesis of a new type of *tris*‐chelating pyridine‐*N*‐oxide based ligands [H_3_(pyO)_3_L]X. Coordination of zinc(II) ions leads to structurally interesting cyclic coordination oligomers which serve as hosts for fullerenes C_60_ and C_70_. The inclusion of fullerenes was observed by single‐crystal X‐ray diffractometry and NMR spectroscopy, and was validated using computations. These metallacycles are robust and show no sign of decomposition. Although computations on such large systems are challenging, we were able to model the C_60_/C_70_ encapsulated metallacycles and even predict spectroscopic data, which are in strong agreement with the experimental results. Electrostatic potential maps reveal that the positive charges of the cavity cores can be tuned by the peripheral halide co‐ligands. These computations help to provide a deeper understanding of host‐guest interaction in these metallacycles. Similar computations will no doubt be useful for the rational design of host molecules with other cargo. In the future, these complexes could serve as container molecules transporting important cargo.2f, 20

## Experimental Section

Experimental methods, synthesis, computational details and results can all be found in the Supporting Information.

CCDC https://www.ccdc.cam.ac.uk/services/structures?id=doi:10.1002/chem.201905390 (**3**, **5**‐Cl, **6**, **7**, **8**, C_60_⊂**9**, C_70_⊂**9**, respectively), contain the supplementary crystallographic data for this paper. These data are provided free of charge by http://www.ccdc.cam.ac.uk/.

## Conflict of interest

The authors declare no conflict of interest.

## Supporting information

As a service to our authors and readers, this journal provides supporting information supplied by the authors. Such materials are peer reviewed and may be re‐organized for online delivery, but are not copy‐edited or typeset. Technical support issues arising from supporting information (other than missing files) should be addressed to the authors.

Supplementary
