# Supplementary material for: Supramolecular Metallacycles and Their Binding of Fullerenes
Source: Chemistry. 2020 Mar 5;26(16):3609–13. doi: 10.1002/chem.201905390 (PMC7155124; doi:10.1002/chem.201905390)
Supplement: Supplementary file 1 — Supplementary [file CHEM-26-3609-s001.pdf]

CHEMISTRY
A European Journal

Supporting Information

Supramolecular Metallacycles and Their Binding of Fullerenes

Christian R. Göb,[a] Andreas Ehnbom,[b] Lisa Sturm,[a] Yoshito Tobe,[c] and Iris M. Oppel*[a]

chem_201905390_sm_miscellaneous_information.pdf

Contents

Experimental **2**

Synthesis **3**

Synthesis of 2-(1,3-dioxolane-2-yl)-pyridine (**1**) 3

Synthesis of 2-(1,3-dioxolane-2-yl)-pyridine-N-oxide (**2**) 3

Synthesis of 2-formylpyridine-N-oxide (**3**) 4

Synthesis of *TAG*-Cl (**4**-Cl) 6

Synthesis of *TAG*-NCS (**4**-NCS) 6

Synthesis of *TAG*-BF_4 (**4**-BF_4) 6

Synthesis of $[H_3(pyO)_3L]Cl$ (**5**-Cl) 6

Synthesis of $[H_3(pyO)_3L]NCS$ (**5**-NCS) 7

Synthesis of $[H_3(pyO)_3L]BF_4$ (**5**-BF_4) 8

Synthesis of $[Zn_{24}Cl_{24}\{(pyO)_3L\}_{12}]$ (**6**) 8

Synthesis of $[Zn_{24}Br_{24}\{(pyO)_3L\}_{12}]$ (**7**) 9

Synthesis of $[Zn_{24}(NCS)_{16}(O_2CH)_8\{(pyO)_3L\}_{12}]$ (**8**) 11

Synthesis of $C_{60} \subset$ **9** 13

Synthesis of $C_{70} \subset$ **9** 14

Synthesis of zinc formate 15

Synthesis of zinc thiocyanate 15

Computational Details **16**

Computational details: geometry optimizations: obtaining a wavefunction 16

Computational details: dispersion corrections 18

Computational details: validation of methodology by crystal structure overlays 18

Computational details: electrostatic potential plots & frontier orbitals 20

Computational details: NMR computations 20

Computational Results **21**

Computational results: interactions of $C_{60/70}$ with metallacycles 21

Computational results: electrostatic potential plots & frontier orbitals 25

Computational results: NMR computations of **9** vs. free C_{60}, and **6** 27

Computational results: IR of **6** 32

Literature **33**

Experimental

Chemicals were used as received without further purification. Fullerenes were purchased in highest purity from Iolitec (Heilbronn, Germany). C_{60} for NMR experiments exhibits an enrichment of 25 mol% ^{13}C.

NMR spectra were measured on either a Bruker Avance II-400 or a Bruker Avance III HD at room temperature. The remaining proton signals of the solvents were used to reference the spectra according to tetramethylsilane (0 ppm).

Dynamic light scattering was measured on a Malvern ZetasizerNanoS with a HeNe-laser (λ = 633 nm) at constant scattering angle (θ = 173°) and a quartz cuvette (1 cm). The sample was filtered using a syringe filter (PTFE, 0.45 μm) and measured three times for 100 s.

Elemental analysis (C, H, N) was carried out at the Institute of Organic Chemistry, RWTH Aachen University, by Claudia Schleep on a Heraeus CHNO-Rapid VarioEL.

Electrospray-Ionization mass spectrometry was performed on a ThermoFisher Scientific LTQ-Orbitrap XL at the Institute of Organic Chemistry, RWTH Aachen University, by Claudia Dittmer.

IR spectra were collected on a Nicolet Avatar 360 E.S.P. spectrometer. The samples were prepared as potassium bromide pellets by a hydraulic press.

Single crystal x-ray data were collected on a Bruker Apex-I CCD-diffractometer (Mo-K_{α}, RWTH Aachen University), a Rigaku SuperNova (Cu-K_{α}, Ruhr-Universität Bochum) with Atlas detector, Rigaku XtaLab (Mo-K_{α}, Osaka University) with a Pilatus 3R 200K detector and a Stoe Stadivari with a Pilatus 3R 200K detector (Cu-K_{α}, RWTH Aachen University). The data was processed with the software packages provided by the manufacturers. Space groups were determined with XPREP (1997). The structures were solved either by a direct method with SHELXS (2013/1) or intrinsic phasing with SHELXT (2017/1). The structure refinement was carried out using SHELXL (2018/3) with a least-squares procedure against F^2. Disordered solvent was treated with the SQUEEZE algorithm implemented in the software package PLATON (2016). Hydrogen atoms were calculated at their idealized positions and refined with a riding model. The isotropic displacement factors of hydrogen atoms were set to 1.2 times the displacement factors of the bound atoms.

Synthesis

Synthesis of 2-(1,3-dioxolane-2-yl)-pyridine (**1**)

2-Formylpyridine (11.08 g, 103.5 mmol), ethylene glycol (18.30 g, 294.8 mmol) and p-toluene sulfonic acid monohydrate (0.78 g, 4.1 mmol) were refluxed in toluene (275 mL) with a Dean-Stark apparatus for 24 hours. The toluene phase was separated and concentrated under reduced pressure. The crude product was used for the following reaction step without further purification. Yield: 13.70 g, 90.6 mmol, 88 %.

^{1}H-NMR (400 MHz, C_6D_6): δ = 8.42 (dm, ^3J = 4.8 Hz, ^4J = 0.9 Hz, 1 H), 7.40 (dt, ^3J = 7.7 Hz, ^4J = 1.1 Hz, 1 H), 7.10 (td, ^3J = 7.6 Hz, ^4J = 1.7 Hz, 1 H), 6.64 (ddd, ^3J = 7.5 Hz, ^4J = 4.8 Hz, ^5J = 1.1 Hz, 1 H), 6.03 (s, 1 H), 3.74-3.64 (m, 2 H), 3.54-3.44 (m, 2 H) ppm.

CHN obs./calc.: C = 62.04/63.56 %, H = 6.54/6.00 %, N = 8.18/9.27 %.

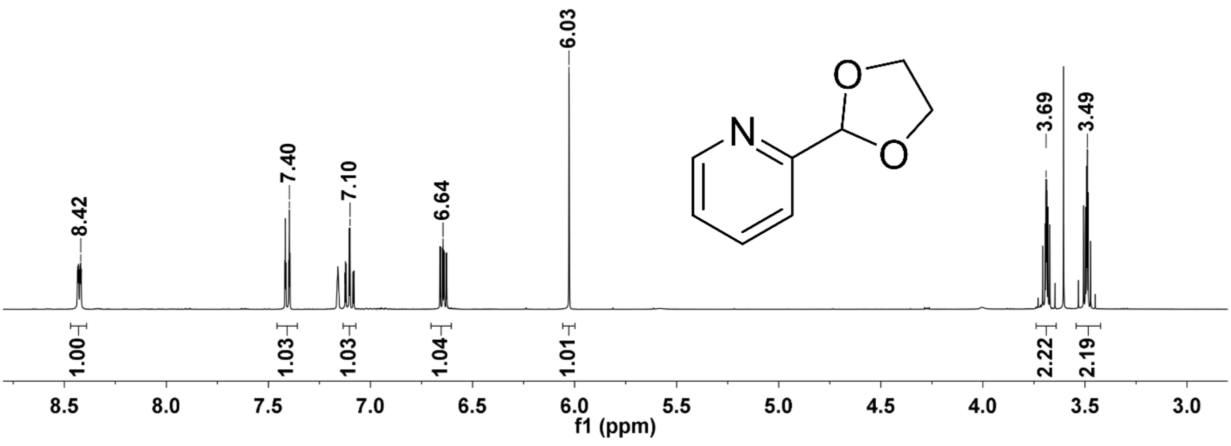

Figure 1: ^{1}H-NMR (C_6D_6) of **1**.

Synthesis of 2-(1,3-dioxolane-2-yl)-pyridine-N-oxide (**2**)

Carbamide peroxide (23.04 g, 243.2 mmol) and phthalic anhydride (13.61 g, 91.9 mmol) were stirred in acetonitrile (100 mL) for 15 minutes. 2-(1,3-dioxolan-2-yl)-pyridine (**1**) (9.26 g, 61.2 mmol) in acetonitrile (150 mL) was added and stirred for another 72 hours. A saturated solution of sodium carbonate was added until pH = 8 and the reaction product was extracted with chloroform (5 x 50 mL). The combined organic layers were washed with brine and dried with sodium sulfate. The solvents were evaporated under reduced pressure to give the product as a pale-yellow oil. Yield: 8.97 g, 53.6 mmol, 88 %.

^{1}H-NMR (400 MHz, $CDCl_3$): δ = 8.28-8.21 (m, 1 H), 7.61-7.53 (m, 2 H), 7.31-7.20 (m, 2 H), 6.37 (s, 1 H), 4.10 (s, 4 H) ppm.

CHN obs./calc.: C = 52.47/57.48 %, H = 5.26/5.43 %, N = 9.44/8.38 %.

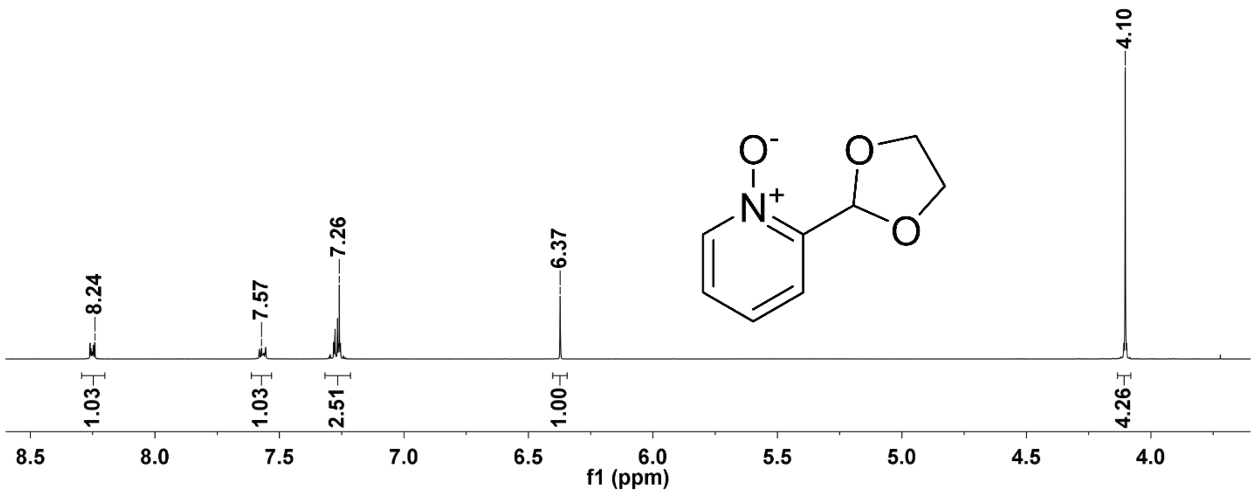

Figure 2: ^{1}H-NMR ($CDCl_3$) of **2**.

Synthesis of 2-formylpyridine-N-oxide (**3**)

2-(1,3-Dioxolan-2-yl)-pyridine-N-oxide (**2**) (15.48 g, 92.6 mmol) was added to hydrochloric acid (20 %, 275 mL) and stirred at 110 °C for 20 minutes. The reaction was quenched in an ice bath and the mixture was neutralized by addition of sodium carbonate. After extraction with chloroform (10 x 200 mL) the combined organic layers were dried with sodium sulfate. The chloroform was evaporated under reduced pressure and the crude product was recrystallized from benzene. Yield: 6.23 g, 50.6 mmol, 55 %.

^{1}H-NMR (400 MHz, $CDCl_3$): δ = 10.62 (s, 1 H), 8.20 (dm, ^{3}J = 6.5 Hz, 1 H), 7.81 (dd, ^{3}J = 7.9 Hz, ^{4}J = 2.1 Hz, 1 H), 7.45 (td, ^{3}J = 7.0 Hz, ^{4}J = 2.2 Hz, 1 H), 7.32 (tm, ^{3}J = 7.7 Hz, 1 H) ppm.

^{13}C-NMR (100 MHz, $CDCl_3$): δ = 185.85, 143.91, 140.46, 130.07, 125.64, 125.26 ppm.

ESI-MS (MeOH): m/z = 124.04 (**3**+H^{+}), 156.07 (**3**+H^{+}+MeOH).

CHN obs./calc.: C = 56.82/58.54, H = 4.47/4.09, N = 10.93/11.38.

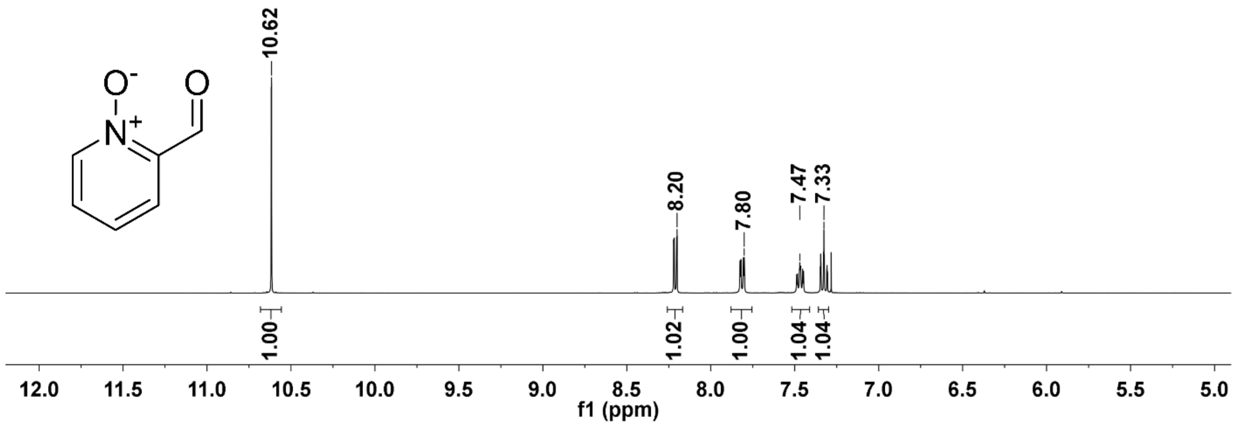

Figure 3: ^{1}H-NMR ($CDCl_3$) of **3**.

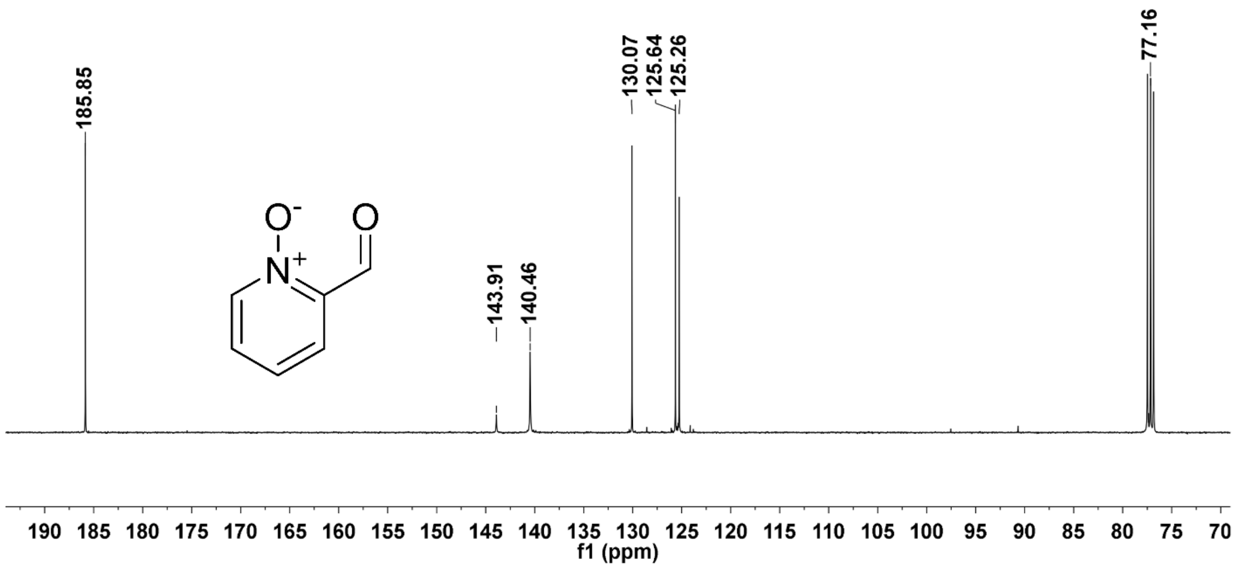

Figure 4: ^{13}C-NMR ($CDCl_3$) of **3**.

Single crystals, suitable for x-ray diffraction experiments, were obtained by slow cooling of a cyclohexane solution from 80°C to room temperature (0.25°C/h).

Crystallographic Data	**3**
Chemical formula	$C_6H_5NO_2$
M [g·mol^{-1}]	123.11
Crystal system	Monoclinic
Space group	$P2_1/n$
Temperature [K]	100(2)
a, b, c [Å]	6.821(2), 4.4598(13), 18.425(6)
α, β, γ [°]	90, 100.438(4), 90
V (Å^3)	551.2(3)
Z	4
λ	Mo K_α
μ [mm^{-1}]	0.11
Crystal size [mm]	0.5 × 0.13 × 0.12
Reflections measured	5386
Reflections independent	1002
Reflections [I > 2σ(I)]	776
R_{int}	0.103
$R[F^2 > 2\sigma(F^2)]$	0.050
$wR(F^2)$	0.129
GOF	1.02
Parameter	82
$\Delta\rho_{max}$, $\Delta\rho_{min}$ [e·Å^{-3}]	0.24, -0.26

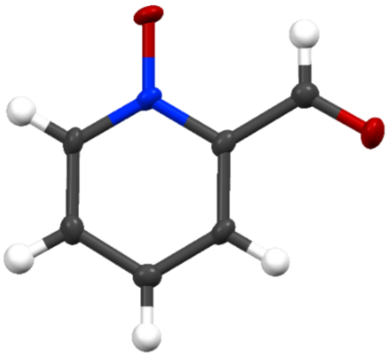

CCDC: 1830922

Synthesis of *TAG*-Cl (**4**-Cl)

Guanidinium chloride (4.78 g, 50 mmol, 1.0 eq) was dissolved in refluxing 1,4-dioxane (20 mL). Hydrazine monohydrate (8.26 g, 0.165 mol, 3.3 eq) in 1,4-dioxane (5 mL) was added dropwise and refluxed for three hours until the evolution of ammonia gas was finished. The product was filtered, washed with acetone, recrystallized from hot EtOH / H_2O (3:2) mixture and dried in vacuum. Yield: 4.93 g, 35.1 mmol, 70 %.

^{1}H-NMR (400 MHz, DMSO-d_6): δ = 8.59 (s, 3 H), 4.48 (s, 6 H) ppm.

CHN obs./calc.: C = 8.91/8.54 %, H = 6.88/6.45 %, N = 61.12/59.78 %.

Synthesis of *TAG*-NCS (**4**-NCS)

Guanidinium isothiocyanate (11.83 g, 100.1 mmol, 1.0 eq) was dissolved in refluxing 1,4 dioxane (30 ml). Hydrazine monohydrate (16.52 g, 330 mmol, 3.3 eq) in 1,4-dioxane (10 ml) was slowly added dropwise and refluxed for four hours until the evolution of ammonia gas was finished. The solvent was evaporated under reduced pressure and the crude product was recrystallized from hot ethanol and dried in vacuum. Yield: 12.65 g, 77.5 mmol, 77 %.

^{1}H-NMR (400 MHz, DMSO-d_6): δ = 8.59 (s, 3 H), 4.48 (s, 6 H) ppm.

CHN obs./calc.: C = 15.03/14.72 %, H = 7.22/5.56 %, N = 57.06/60.08 %.

Synthesis of *TAG*-BF_4 (**4**-BF_4)

Triaminoguanidinium chloride (1000.7 mg, 7.1 mmol, 1.0 eq, **4**-Cl) and silver tetrafluoroborate (1385.7 mg, 7.1 mmol, 1.0 eq) were each dissolved in water and combined under strong stirring. The precipitate of silver chloride was removed by filtration, the filtrate was concentrated under reduced pressure and dried in high vacuum (40 °C). Yield: 1.32 g, 6.9 mmol, 97 %.

^{1}H-NMR (400 MHz, DMSO-d_6): δ = 8.58 (s, 3 H), 4.47 (s, 6 H) ppm.

CHN obs./calc.: C = 7.90/6.26 %, H = 4.88/4.73 %, N = 42.92/43.79 %.

Synthesis of [H_3(pyO)$_3$*L*]Cl (**5**-Cl)

TAG-Cl (**4**-Cl) (736.6 mg, 5.24 mmol, 1.0 eq) and 2-formylpyridine-N-oxide (**3**) (2.00 g, 16.25 mmol, 3.1 eq) were dissolved in water (40.0 mL) and ethanol (20.0 mL) and stirred for three days. The product was separated by filtration, washed with diethyl ether and dried in vacuum. Yield: 2.34 g, 5.13 mmol, 98 %.

^{1}H-NMR (400 MHz, DMSO-d_6): δ = 12.01 (s, NH), 9.12 (s, 1 H), 8.36 (d, ^3J = 6.6 Hz, 1 H), 8.31 (dd, ^3J = 6.5 Hz, ^4J = 1.0 Hz, 1 H), 7.55-7.40 (m, 2 H) ppm.

ESI-MS (MeOH): 420.15 m/z ([H_3(pyO)$_3$*L*]$^+$).

CHN obs./calc.: C = 42.05/50.06 %, H = 5.42/3.98 %, N = 23.46/27.65 %.

Single crystals, suitable for x-ray diffraction experiments, were obtained by solvent evaporation of a DMSO solution at 40°C under a stream of nitrogen gas.

Crystallographic data	**4**-Cl
Chemical formula	$C_{23}H_{30}ClN_9O_5S_2$
M [g·mol^{-1}]	612.12
Crystal system	Monoclinic
Space group	$P2_1/n$
Temperature [K]	100(2)
a, b, c [Å]	9.59406(10), 12.58809(15), 23.1568(4)
α, β, γ [°]	90, 94.6089(12), 90
V (Å^3)	2787.62(6)
Z	4
λ	Cu K_α
μ [mm^{-1}]	3.06
Crystal size [mm]	0.15 × 0.14 × 0.11
Reflections measured	23068
Reflections independent	5723
Reflections [$I > 2\sigma(I)$]	5238
R_{int}	0.020
$R[F^2 > 2\sigma(F^2)]$	0.037
$wR(F^2)$	0.097
GOF	1.14
Parameter	365
$\Delta\rho_{max}$, $\Delta\rho_{min}$ [e·Å^{-3}]	0.32, -0.36

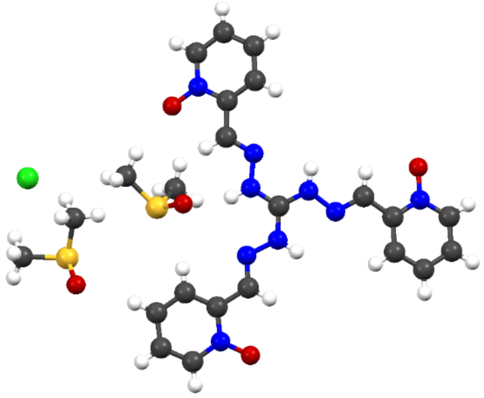

CCDC: 1830936

Synthesis of [$H_3(pyO)_3$*L*]NCS (**5**-NCS)

TAG-NCS (**4**-NCS) (128.4 mg, 0.79 µmol, 1.0 eq) and 2-formylpyridine-N-oxide (**3**) (299.7 mg, 2.43 mmol, 3.1 eq) were dissolved in water (7.0 mL) and ethanol (3.5 mL) and stirred for three days. The product was separated by filtration, washed with diethyl ether and dried in vacuum. Yield: 346.2 mg, 0.72 mmol, 92 %.

^{1}H-NMR (400 MHz, DMSO-d_6): δ = 12.30 (s, NH), 9.20 (s, 1 H), 8.38 (dd, ^3J = 7.7 Hz, ^4J = 1.8 Hz, 1 H), 8.34 (dd, ^3J = 6.3 Hz, ^4J = 0.8 Hz, 1 H), 7.49 (td, ^3J = 7.0 Hz, ^4J = 2.4 Hz, 1 H), 7.48 (td, ^3J = 7.6 Hz, ^4J = 1.0 Hz, 1 H) ppm.

CHN obs./calc.: C = 46.55/50.20 %, H = 4.29/3.79 %, N = 27.28/29.27 %.

Synthesis of $[H_3(pyO)_3L]BF_4$ (**5**-BF_4)

TAG-BF_4 (**4**-BF_4) (251.3 mg, 1.31 mmol, 1.0 eq) and 2-formylpyridine-N-oxide (**3**) (500.8 mg, 4.07 mmol, 3.1 eq) were dissolved in water (10.0 mL) and ethanol (5.0 mL) and stirred for three days. The product was separated by filtration, washed with diethyl ether and dried in vacuum. Yield: 549.7 mg, 1.08 mmol, 82 %.

^{1}H-NMR (400 MHz, DMSO-d_6): δ = 12.30 (s, NH), 9.20 (s, 1 H), 8.38 (dd, ^3J = 7.7 Hz, ^4J = 1.8 Hz, 1 H), 8.34 (dd, ^3J = 6.3 Hz, ^4J = 0.8 Hz, 1 H), 7.49 (td, ^3J = 7.0 Hz, ^4J = 2.4 Hz, 1 H), 7.48 (td, ^3J = 7.6 Hz, ^4J = 1.0 Hz, 1 H) ppm.

CHN obs./calc.: C = 39.25/44.99 %, H = 4.49/3.58 %, N = 22.13/24.85 %.

Synthesis of $[Zn_{24}Cl_{24}\{(pyO)_3L\}_{12}]$ (**6**)

$[H_3(pyO)_3L]Cl$ (**5**-Cl) (10.0 mg, 21.9 µmol, 1.0 eq) and zinc formate (10.2 mg, 65.8 µmol, 3.0 eq) were covered with dimethylformamide (1.0 mL). The reaction mixture was allowed to stand in the fridge (4°C) for one week. A few orange octahedral crystals could be collected between amorphous precipitate (major reaction product).

Crystallographic data	**6**
Chemical formula	$C_{228}H_{180}Cl_{24}N_{108}O_{36}Zn_{24}$
M [g·mol^{-1}]	7428.59
Crystal system	Monoclinic
Space group	C2/c
Temperature [K]	160(2)
a, b, c [Å]	28.5716(5), 32.7651(7), 48.6449(10)
α, β, γ [°]	90, 91.218(2), 90
V (Å^3)	45528.7(16)
Z	4
λ	Mo K_α
μ [mm^{-1}]	1.43
Crystal size [mm]	0.41 × 0.21 × 0.18
Reflections measured	188305
Reflections independent	40030
Reflections [I > 2σ(I)]	24466
R_{int}	0.058
$R[F^2 > 2\sigma(F^2)]$	0.116
$wR(F^2)$	0.412
GOF	1.55
Parameter	745
$\Delta\rho_{max}$, $\Delta\rho_{min}$ [e·Å^{-3}]	3.13, -0.94

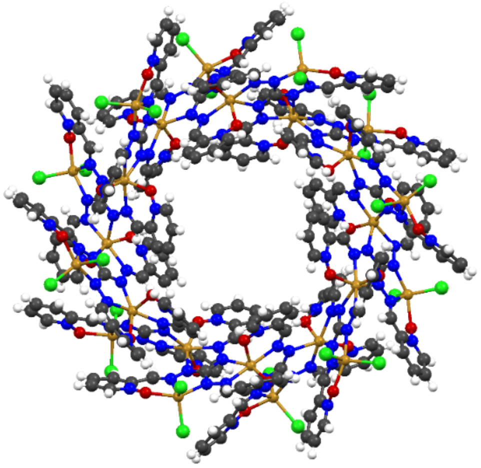

CCDC 1830997

Synthesis of $[Zn_{24}Br_{24}\{(pyO)_3L\}_{12}]$ (**7**)

$[H_3(pyO)_3L]BF_4$ (**5**-BF_4) (40.0 mg, 78.9 µmol, 1.0 eq), zinc bromide (53.2 mg, 236.2 µmol, 3.0 eq) and sodium formate (16.0 mg, 235.3 µmol, 3.0 eq) were covered with dimethyl-formamide (2.0 mL). The reaction mixture was allowed to stand at room temperature. Orange crystals were collected after one month. Yield: 74.2 mg, 6.71 µmol, 8.5 %, calculation based on SQUEEZE results in PLATON (2016) for [**7** · 44 DMF].

CHN obs./calc.: C = 32.32/32.23 %, H = 2.79/2.14 %, N = 16.96/17.81 %.

Crystallographic data	**7**
Chemical formula	$C_{228}H_{180}Br_{24}N_{108}O_{36}Zn_{24}$
M [g·mol^{-1}]	8495.48
Crystal system	Monoclinic
Space group	C2/c
Temperature [K]	100(2)
a, b, c [Å]	29.005(6), 33.325(7), 48.848(10)
α, β, γ [°]	91.78(3)
V (Å^3)	47195(16)
Z	4
λ	Cu K_α
μ [mm^{-1}]	4.11
Crystal size [mm]	0.29 × 0.22 × 0.07
Reflections measured	125507
Reflections independent	29537
Reflections [I > 2σ(I)]	14716
R_{int}	0.115
R[F^2 > 2σ(F^2)]	0.149
wR(F^2)	0.400
GOF	2.14
Parameter	740
$\Delta\rho_{max}$, $\Delta\rho_{min}$ [e·Å^{-3}]	1.41, -0.93

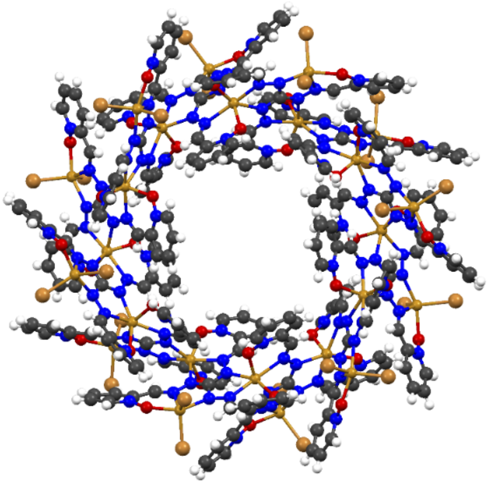

CCDC 1851696

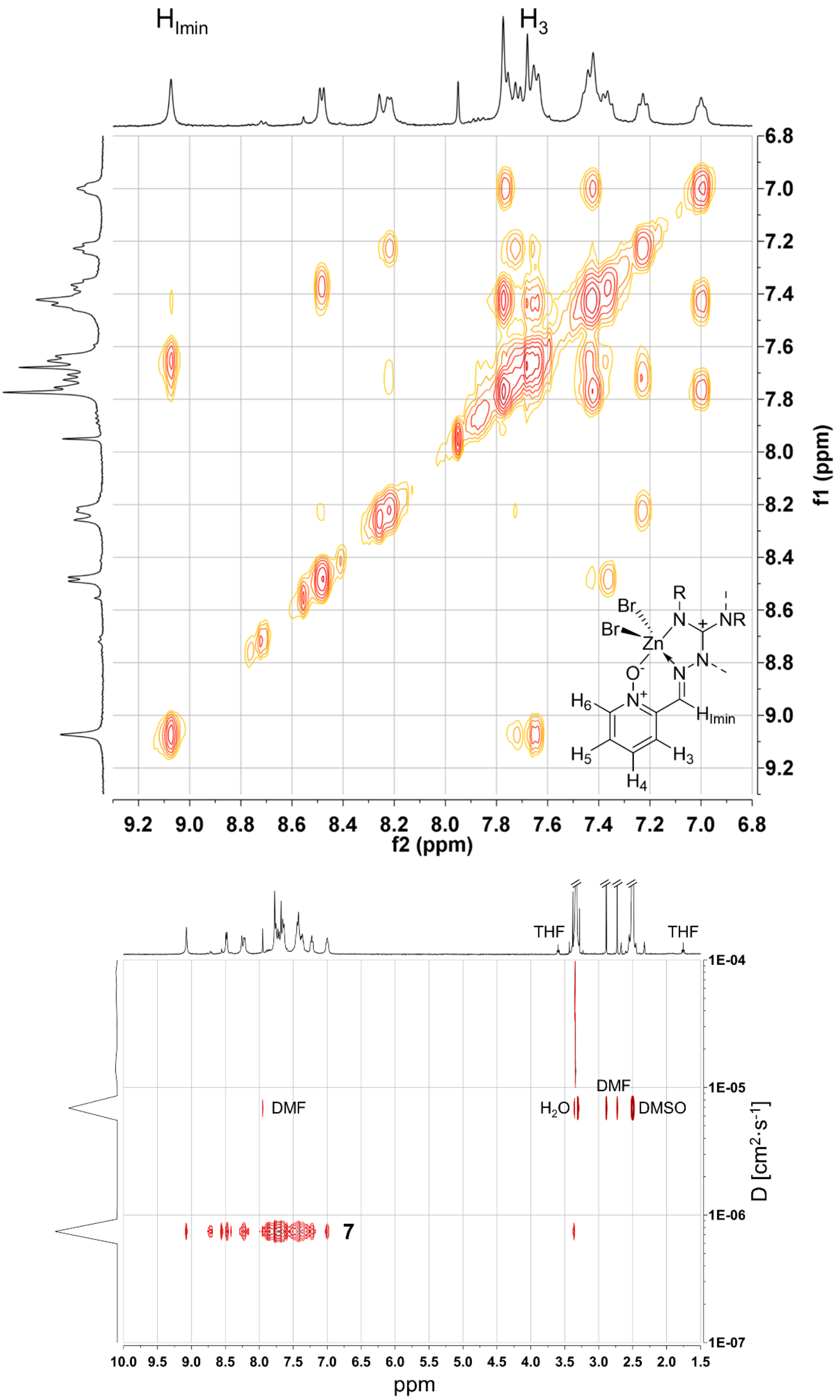

Figure 5: ^{1}H-^{1}H-NOESY-NMR and ^{1}H-DOSY-NMR of **7** in DMSO-d$_6$.

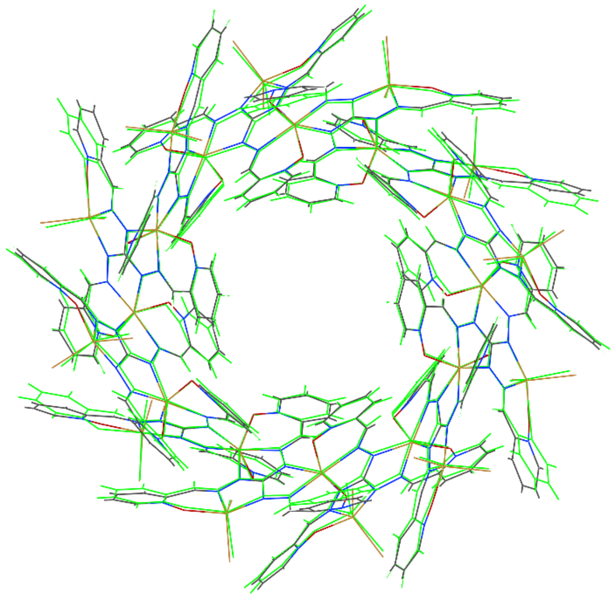

Figure 6: Superposition of crystal structures of **7** and **6** (green).

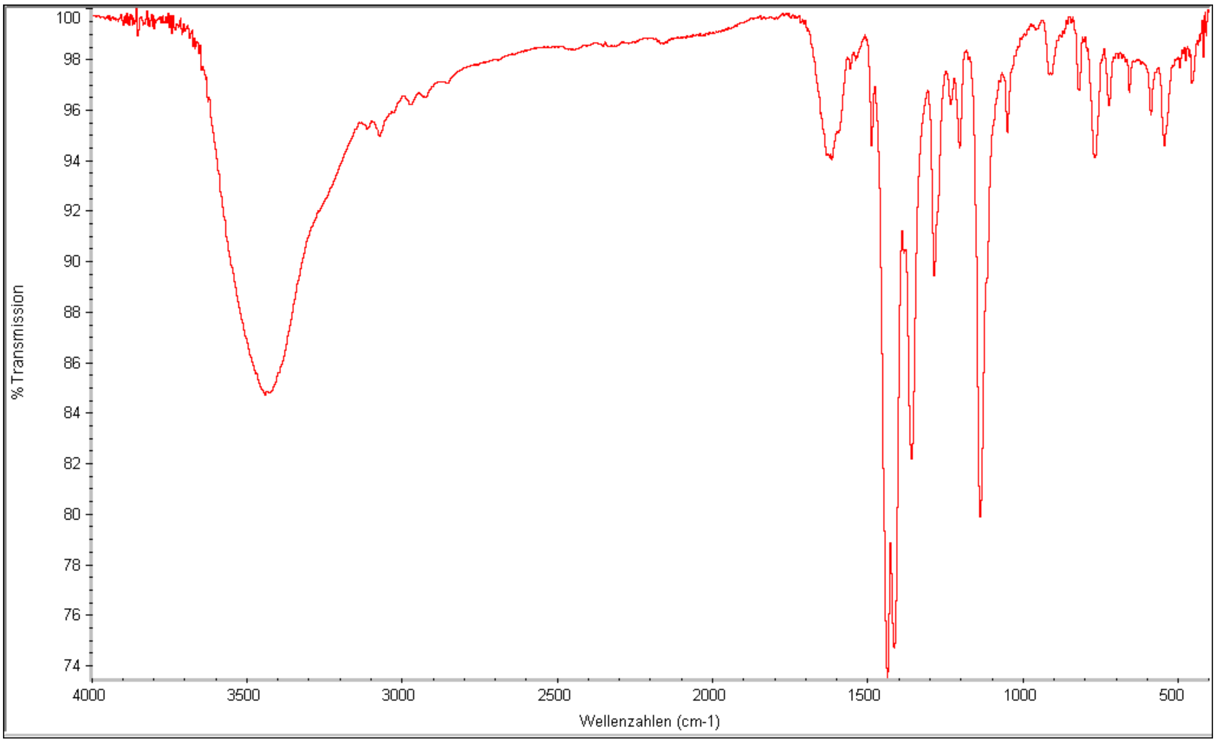

Figure 7: IR (KBr) of **7**.

Synthesis of $[Zn_{24}(NCS)_{16}(O_2CH)_8\{(pyO)_3L\}_{12}]$ (**8**)

$[H_3(pyO)_3L]$NCS (**5**-NCS) (10.0 mg, 20.9 µmol, 1.0 eq), zinc thiocyanate (11.4 mg, 62.7 µmol, 3.0 eq) and sodium formate (4.3 mg, 62.7 µmol, 3.0 eq) were covered with dimethylformamide (1.0 mL). The reaction mixture was allowed to stand at room temperature (22°C) for one week and orange octahedral crystals could be collected. Yield: 10.3 mg, 1.0 µmol, 59 %, calculation based on SQUEEZE results in PLATON (2016) for [**8** · 28 DMF].

CHN obs./calc.: C = 34.44/38.48 %, H = 2.89/2.41 %, N = 18.78/22.08 %.

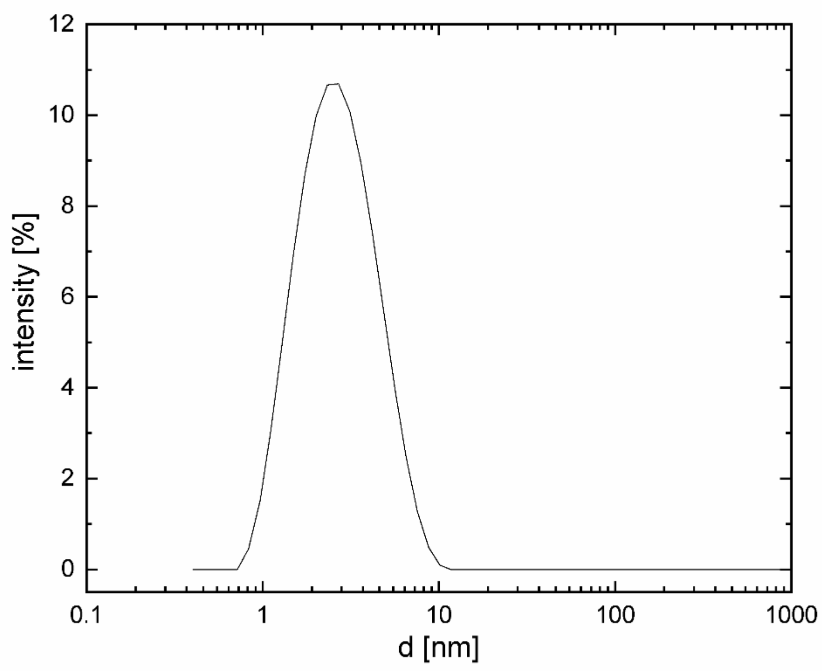

Figure 8: DLS of **8** (DMSO, 1 mg·mL^{-1}): d = 2.9±1.5 nm.

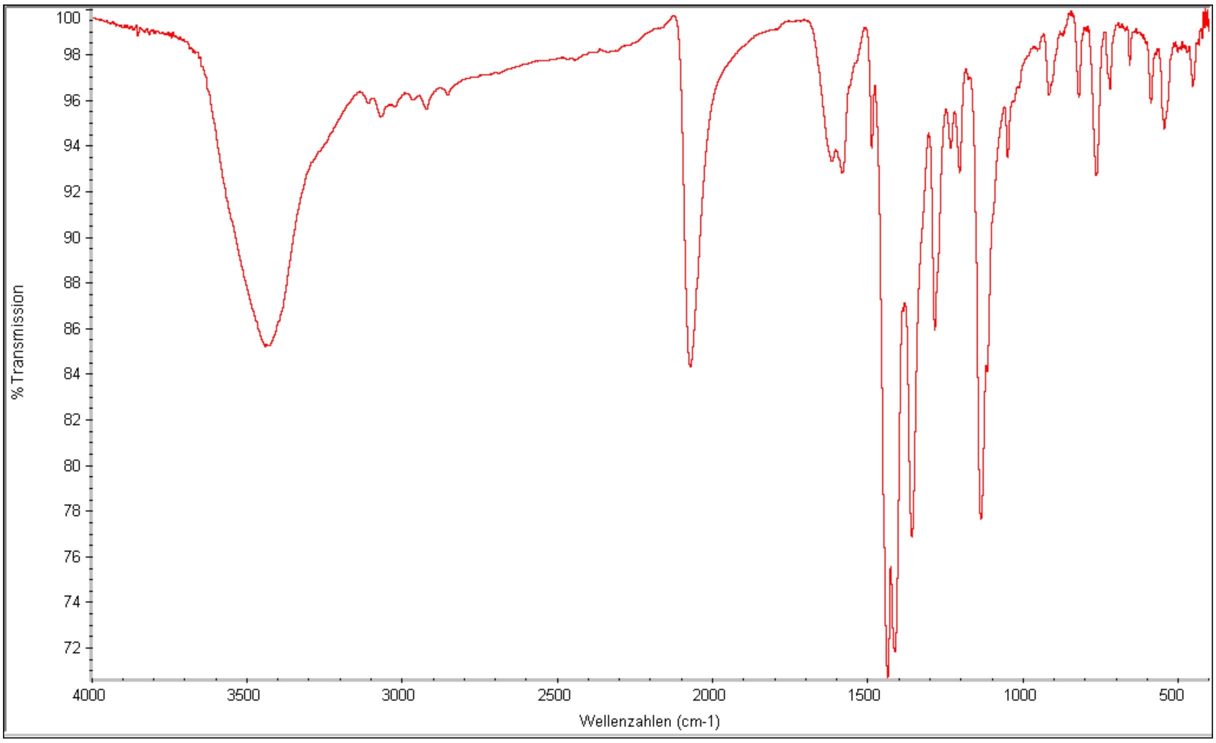

Figure 9: IR (KBr) of **8**.

Crystallographic data	**8**
Chemical formula	$C_{252}H_{188}N_{124}O_{52}S_{16}Zn_{24}$
M [g·mol^{-1}]	7867.17
Crystal system	Tetragonal
Space group	$P\bar{4}2_1c$
Temperature [K]	100(2)
a, b, c [Å]	29.1575(8), 29.1575(8), 26.1277(13)
α, β, γ [°]	90, 90, 90
V (Å^3)	22212.7(16)
Z	2
λ	Mo K_α
μ [mm^{-1}]	1.41
Crystal size [mm]	0.16 × 0.13 × 0.1
Reflections measured	256333
Reflections independent	21322
Reflections [I > 2σ(I)]	16053
R_{int}	0.227
$R[F^2 > 2\sigma(F^2)]$	0.066
$wR(F^2)$	0.177
GOF	1.02
Parameter	946
$\Delta\rho_{max}$, $\Delta\rho_{min}$ [e·Å^{-3}]	0.59, -0.53
Flack-Parameter	0.041(10)

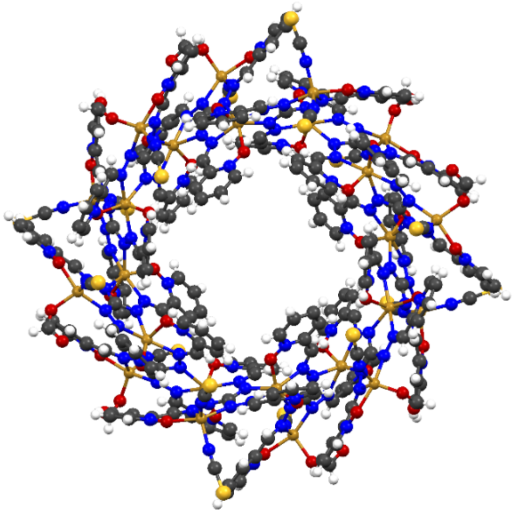

CCDC 1831512

Synthesis of $C_{60} \subset$ **9**

[$H_3(pyO)_3$*L*]NCS (**5**-NCS) (10.0 mg, 20.9 µmol), zinc thiocyanate (11.4 mg, 62.7 µmol) and sodium formate (4.3 mg, 62.7 µmol) were covered with dimethylformamide (1.0 mL). C_{60} (7.6 mg, 10.5 µmol) was dissolved in toluene and added to the reaction mixture, which was allowed to stand at room temperature (22°C) for one week. Dark red octahedral crystals could be collected. Yield: 14.0 mg, 1.3 µmol, 75 %, calculation based on SQUEEZE results in PLATON (2016) for [$C_{60} \subset$ **9** · 24 DMF].

CHN obs./calc.: C = 38.03/43.64 %, H = 2.73/2.21 %, N = 19.00/20.22 %.

Crystallographic treatment of C_{60}: Due to its position on a four-fold rotoinversion axis, C_{60} was treated as a fragment from the *Idealized Molecular Geometry Library* and occupied by ¼.[s19] Additionally a variable for the isotropic temperature factor was used.

Crystallographic data	$C_{60}\subset$**9**
Chemical formula	$C_{312}H_{184}N_{128}O_{44}S_{20}Zn_{24}$
M [g·mol^{-1}]	8640.08
Crystal system	tetragonal
Space group	$P\bar{4}2_1c$
Temperature [K]	100(2)
a, b, c [Å]	29.6054(10), 29.6054(10), 25.8990(19)
α, β, γ [°]	90, 90, 90
V (Å^3)	22700(2)
Z	2
λ	Mo K_α
μ [mm^{-1}]	1.40
Crystal size [mm]	0.11 × 0.10 × 0.10
Reflections measured	107700
Reflections independent	5419
Reflections [I > 2σ(I)]	4915
R_{int}	0.110
R[F^2 > 2σ(F^2)]	0.060
wR(F^2)	0.211
GOF	1.02
Parameter	398
$\Delta\rho_{max}$, $\Delta\rho_{min}$ [e·Å^{-3}]	0.58, -0.38
Flack-Parameter	0.044(8)

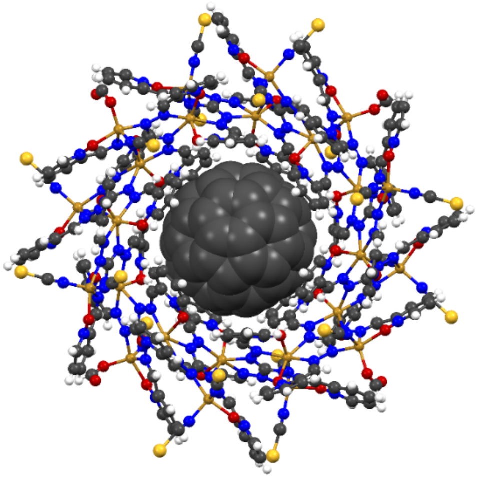

CCDC 1831515

Synthesis of $C_{70} \subset$ **9**

[H_3(pyO)$_3$*L*]NCS (**5**-NCS) (5.0 mg, 10.5 µmol), zinc thiocyanate (5.7 mg, 31.4 µmol) and sodium formate (2.1 mg, 31.4 µmol) were covered with dimethylformamide (0.45 mL). C_{70} (4.4 mg, 5.2 µmol) in toluene (0.05 mL) was added and the reaction mixture was allowed to stand at room temperature (22°C) for one week. Dark red octahedral crystals could be collected. Yield: 6.3 mg, 8.7 µmol, 69 %, calculation based on SQUEEZE results in PLATON (2016) for [$C_{70} \subset$ **9** · 24 DMF].

Crystallographic treatment of C_{70}: Due to its position on a four-fold rotoinversion axis, C_{70} was treated as a fragment from D. Tománek *et al.* and occupied by ¼.[s20] Additionally a variable for the isotropic temperature factor was used.

Crystallographic data	C_{70}⊂**9**
Chemical formula	$C_{322}H_{184}N_{128}O_{44}S_{20}Zn_{24}$
M [g·mol^{-1}]	8760.19
Crystal system	tetragonal
Space group	$P\bar{4}2_1c$
Temperature [K]	100(2)
a, b, c [Å]	29.638(4), 29.638(4), 25.951(5)
α, β, γ [°]	90, 90, 90
V (Å^3)	22796(8)
Z	2
λ	Cu K_α
μ [mm^{-1}]	2.76
Crystal size [mm]	0.12 × 0.10 × 0.08
Reflections measured	186908
Reflections independent	14304
Reflections [I > 2σ(I)]	7413
R_{int}	0.243
R[F^2 > 2σ(F^2)]	0.111
wR(F^2)	0.333
GOF	1.12
Parameter	923
$\Delta\rho_{max}$, $\Delta\rho_{min}$ [e·Å^{-3}]	1.83, -1.64
Flack-Parameter	0.5

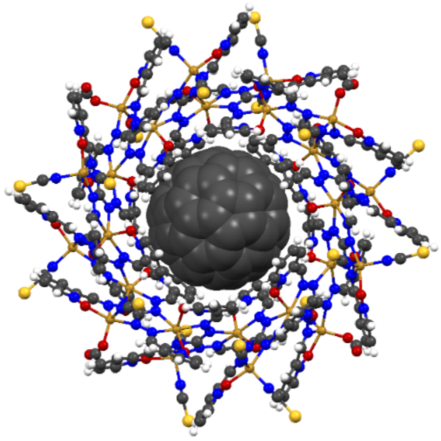

CCDC 1831516

Synthesis of zinc formate

Basic zinc carbonate (58 % Zn, 3.45 g, 30.6 mmol Zn) was dissolved in a mixture of water (50 mL) and formic acid (6.9 mL, 183.5 mmol). After refluxing for two hours, water and excess formic acid were distilled off and the residue was dried in high vacuum at 140°C for 18 hours. Yield: 4.28 g, 27.5 mmol, 90 %.

Synthesis of zinc thiocyanate

Zinc sulfate heptahydrate (9.35 g, 32.5 mmol) and barium thiocyanate trihydrate (10.00 g, 32.5 mmol) were each dissolved in 20 mL of water and combined with strong stirring. The reaction mixture was stirred for 24 hours and filtered. The filtrate was concentrated in vacuum and dried at 80°C in high vacuum. Yield: 5.51 g, 30.4 mmol, 94 %.

Computational Details

Computational details: geometry optimizations: obtaining a wavefunction

The computations were run using the Gaussian16 software suite, which by default employs an ultrafine grid (99,590) for improved accuracy.[s1] The computations were run on an HPRC cluster with 28 cores.[s2] A GGA-type functional (BP86)[s3] and a hybrid functional (B3LYP)[s3a,s4] were tested to obtain the wavefunction. The GGA-type functional BP86 was found to converge the wavefunction best.

Pople type (all-electron) basis sets were tested with and without polarization functions and diffuse functions.[s5,s6] There are restrictions on which electronic structure methods and basis set can be used for these large systems. An empty metallacycle contains 600/600/656 atoms and 4152/3720/3960 electrons (Br/Cl/NCS). The computational code could not perform frequency calculations for these systems. Thus, neither the exact nature of all optimized structures nor the thermodynamic parameters could be confirmed. Qualitative arguments can still be inferred from the computed energies. There is only one instance of a C_2-symmetrized Cl-metallacycle (**6**), which could undergo a frequency analysis. It showed all modes to be real and yielded an IR with similar characteristic peaks to an experimental chloride metallacycle, although the calculation were performed in gas phase (see Figure 25).

A comparison of the 6-31g[s5] vs. 6-311g[s6] energy curves, when moving a C_{60} guest out of a chloride metallacycle, using the BP86 functional and GD3BJ dispersion corrections, is shown in Figure 10.[s7] There is a negligible difference in the energetic profile when using a double- or triple-ζ-quality basis set, even when SMD solvent corrections[s8] in benzene ("•") are used compared to gas phase computations ("•,•"). The energetic profile does not drastically change although the barrier for C_{60} entering the metallacycle does increase when solvent corrections are added.

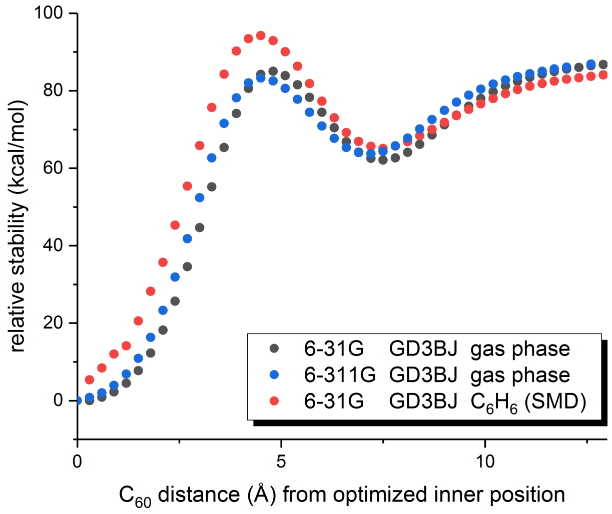

Figure 10: Single-point calculations using different computational settings with a stepwise extrusion of C_{60} from the fully optimized structure of a Cl-metallacycle containing C_{60} (at 0 Å).

No changes in convergence criteria were required for any of the calculations and no restrictive symmetry keywords were imposed during optimization. High symmetry in a molecule can often be beneficial when optimizing large structures as the system (wavefunction) becomes smaller, leading to faster computations. However, high symmetry may lead to degeneracy problems (orbitals with similar energies), which give rise to convergence problems. The command "Symmetry=None" was used in these optimizations to more quickly obtain a converged wavefunction in most cases. Only one of the structures (Figure 11) was kept in a high symmetry point group in order to compute NMR-calculations of an empty metallacycle (**6**). Another version of **6** with C_2-symmetry was used to perform a frequency calculation.

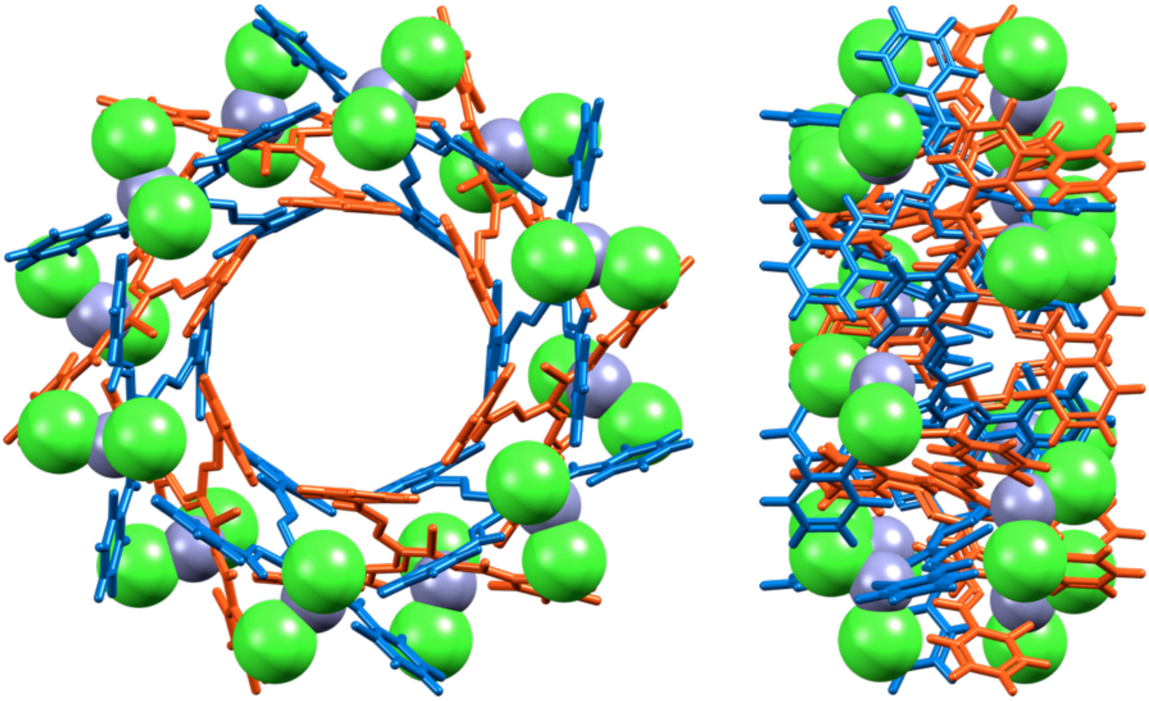

Figure 11: An empty metallacycle (**6**) with the highest possible symmetry, point group: S_{12}. The ligands, which are related by symmetry, are shown in the same color. A 90° relationship is present between the left and right depiction of **6**. The gray and green spheres indicate Zn(II) and Cl^- units, respectively.

Computational details: dispersion corrections

The main interaction type between guest molecules (C_{60} or C_{70}) and the host (metallacycle) is dispersion.[s9] Classical functionals (e.g. BP86, B3LYP) do not sufficiently include dispersion and must therefore be corrected in order to obtain accurate results. Several of Grimme's dispersion corrections were examined (Figure 12).[s7,s10,s11] The older "GD2"[s10] ("◂") and "GD3"[s11] ("◆") corrections have both a tendency to overcorrect the dispersion interactions. Thus, the most recent "GD3BJ" D3 version of Grimme's dispersion function which includes Becke-Johnson damping was used throughout the computations ("•").[s7] If no dispersion corrections are implemented, there are no favorable interactions between C_{60} and the metallacycle (Figure 12, "•").

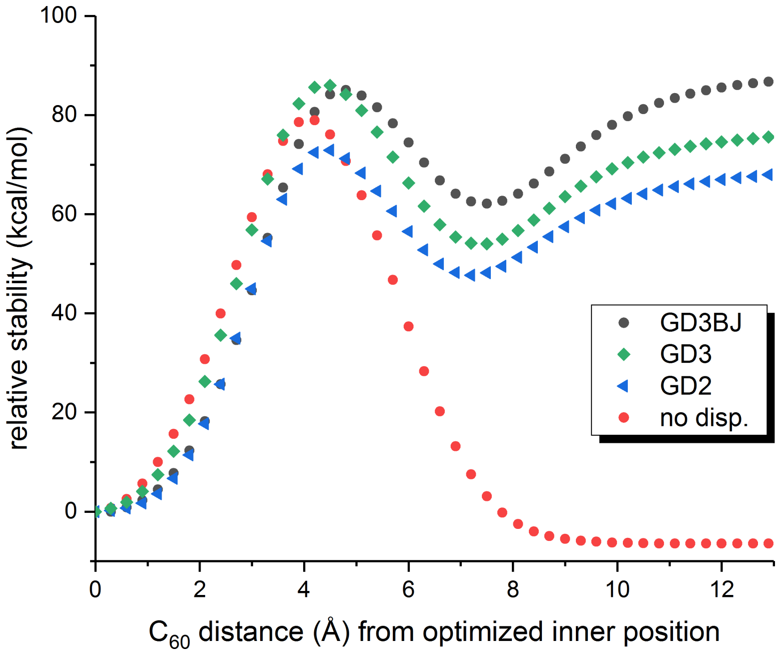

Figure 12: Comparison of relative stability using different dispersion corrections. Model system is a C_{60}-filled **6**. The C_{60} is translated out of the metallacycle in small increments using single-point calculations.

Computational details: validation of methodology by crystal structure overlays

Different single crystal structures in different space groups were used as input structures during optimizations. The metallacycles were challenging to compute because of their size. But since some of them had an inherent symmetry, the wavefunction could be converged. However, the computational code could not generate the frequency calculations as almost 8000 primitive functions were handled and it can only handle up to approx. 2000. The number of maximum SCF-cycles for convergence was increased to 500. The optimized structures from the computations and the crystallographically characterized species were overlaid using the software Mercury (Figure 13-15).[s12] The molecules were superimposed by using the built-in "Molecular Overlay" function which minimizes the RMSD upon overlaying the two structures. Figures 13-15 show three different types of structures overlaid with

excellent structural agreement between computed and experimental data. The empty chloride metallacycle **6** (C2/c space group, element colors) vs. the computed structure (orange) resulted in an RMSD of 0.97 showing overlaid in Figure 13. The empty bromide metallacycle **7** (C2/c space group, element colors) vs. the computed structure (orange) resulted in an RMSD of 0.86 showing overlaid in Figure 14. The empty isothiocyanate metallacycle **8** ($P\bar{4}2_1c$ space group, element colors) vs. the computed structure (orange) resulted in an RMSD of 0.39 showing overlaid in Figure 15.

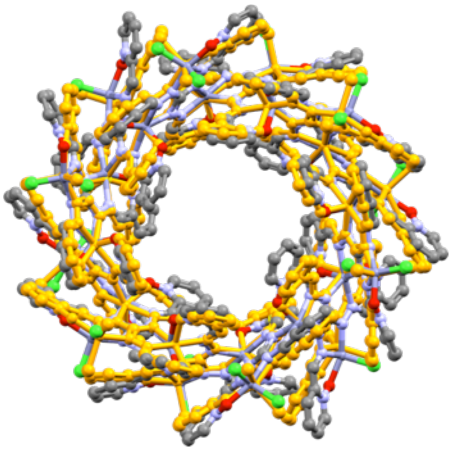

Figure 13: Comparison of computed geometry optimized structure (orange) and XRD of **6**.

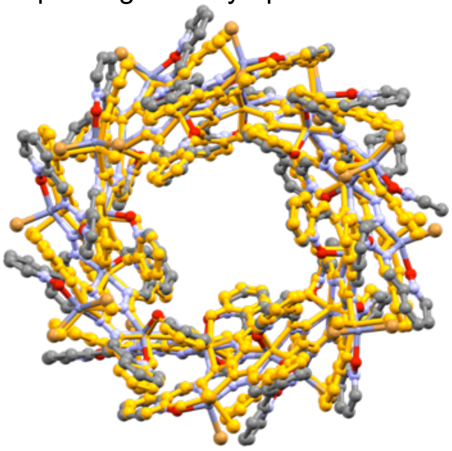

Figure 14: Comparison of computed geometry optimized structure (orange) and XRD of **7**.

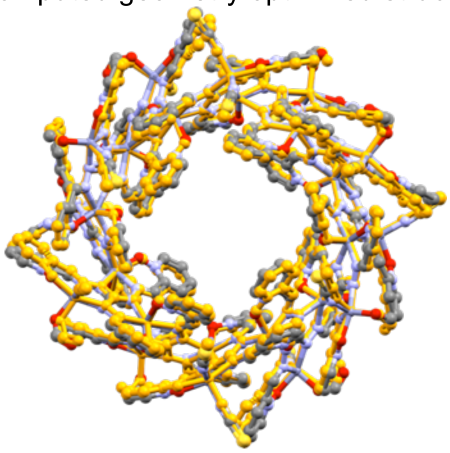

Figure 15: Comparison of computed geometry optimized structure (orange) and XRD of **8**.

Computational details: electrostatic potential plots & frontier orbitals

All plots were rendered using a density of ρ = 0.004 e/au^3 (0.0004 for filled metallacycles) and surfaces were rendered with a fixed isovalue of 0.01 to be able to cross-compare all data. The surface scales were fixed to a range of –0.065 to 0.065 au. Individual job-files were prepared where the requested memory could be extended in order to run the jobs directly on the cluster as oppose to using the cubegen utility within Gaussview 6.[s13]

Computational details: NMR computations

Computations of NMR chemical shift for the C_{60} assembly lead to multiple peaks for the C_{60} guest. The reason for this is because there is no dynamic behavior in the NMR computations. The computations can be thought of as observing an experimental spectrum of a system acquired at 0 K. Thus, the normal distribution of the C_{60} peaks were taken plotted for the C_{60} free vs. **9**. There is a significant downfield shift of the signal in going from the free to the interacting C_{60} ^{13}C spectra. Because of this naturally occurring tumbling process at room temperature we would expect the metallacycle-contained C_{60} signal to be broader than the free, which is also observed experimentally. There are several relevant computational studies of C_{60} systems detailing how to obtain accurate NMR calculations for analogous systems.[s14] A useful review over common practices and errors in computational NMR computations is also provided.[s15]

Analogous to computing frequency computations, NMR calculations were extra challenging for these large systems. Ideally, a large basis set with plenty of diffuse- and polarization functions [e.g. 6-311++G(d,p)] are required to obtain accurate NMR chemical shift.[s16] These higher-level basis set would not converge and 6-31G was used. The Gauge-Independent Atomic Orbital (GIAO) method[s17] was used to compute the isotropic chemical shift. Spin-spin couplings were also computed adding the command "spinspin".[s13]

Computational Results

Computational results: interactions of $C_{60/70}$ with metallacycles

The computations of the interactions between the metallacycle and C_{60}/C_{70} show qualitatively that C_{70} results in a slightly larger stabilization (Figure 16). At the same time, the barrier for the larger C_{70} to enter the metallacycle is qualitatively larger than C_{60}, which is intuitive due to a larger steric clash with phenyl groups on the metallacycle. Because these calculations (except for 0 Å) are single-point calculations, the geometry of the metallacycle is not relaxed upon any C_{60}/C_{70} movement. This results in disproportionately high barriers (**B**), which would be significantly lower if the geometries of the metallacycle were allowed to relax.

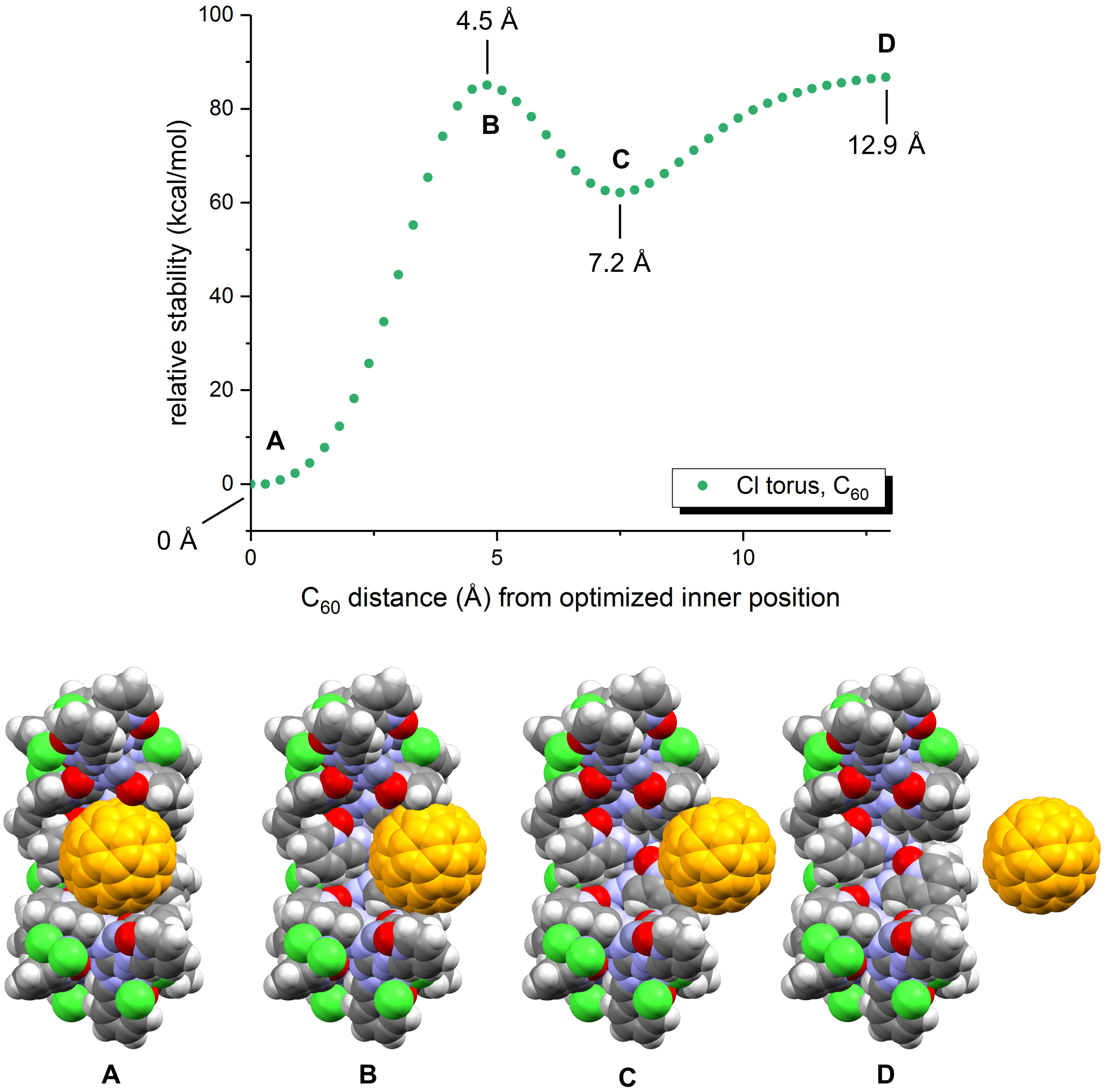

Figure 16: Relative stability of a C_{60} exiting a Cl-metallacycle at the BP86/6-31G level of theory (gas phase) with GD3BJ dispersion corrections. The C_{60} structure is colored orange and only half of the metallacycle is shown for clarity.

There is a local minimum around 7.2 Å from the center of the optimized position of the metallacycles (Cl, Br, and NCS). A high-energy point (possibly a transition state) is found around 4.5 Å from the center of the optimized metallacycle. This high-energy point is observed because the repulsive terms of the C_{60} outcompete the attractive dispersion forces. Furthermore the edge of the metallacycle has groups facing inwards, creating a lip of the entrance, which the C_{60} has to pass. The metallacycle does not have the opportunity to relax to adjust the "entry port" to the incoming $C_{60/70}$ (which otherwise would have been wider) because of the nature of the single-point calculations.

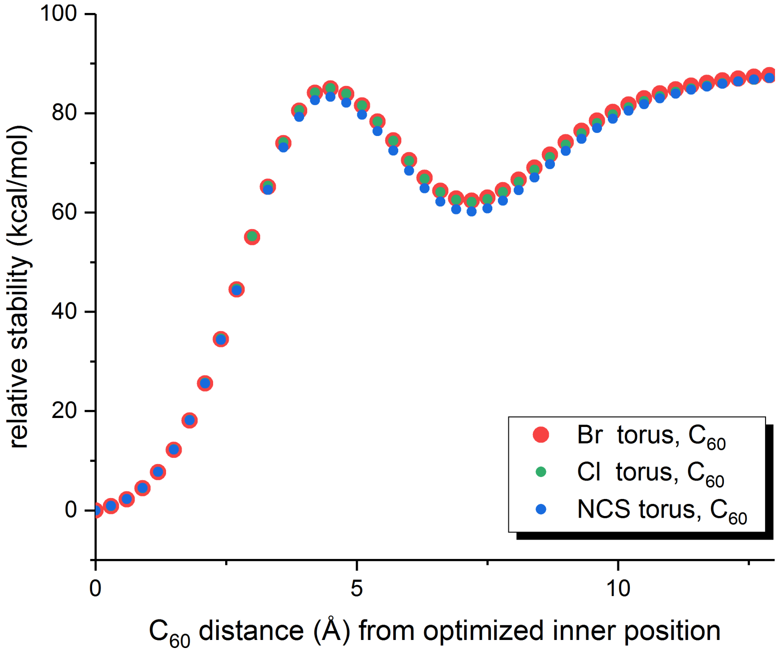

Figure 17: Relative stability of a C_{60} exiting a Cl/Br/NCS metallacycle at the BP86/6-31G level of theory (gas phase) with GD3BJ dispersion corrections.

It is clearly visible from Figure 18 **A_1** and **A_2** that the optimized metallacycle for C_{70} is slightly oval and less spherical in shape. This results in the enclosing of phenyl groups of the metallacycle (shown in yellow) that cause a larger steric repulsion when the C_{70} exits the metallacycle compared to the C_{60}.

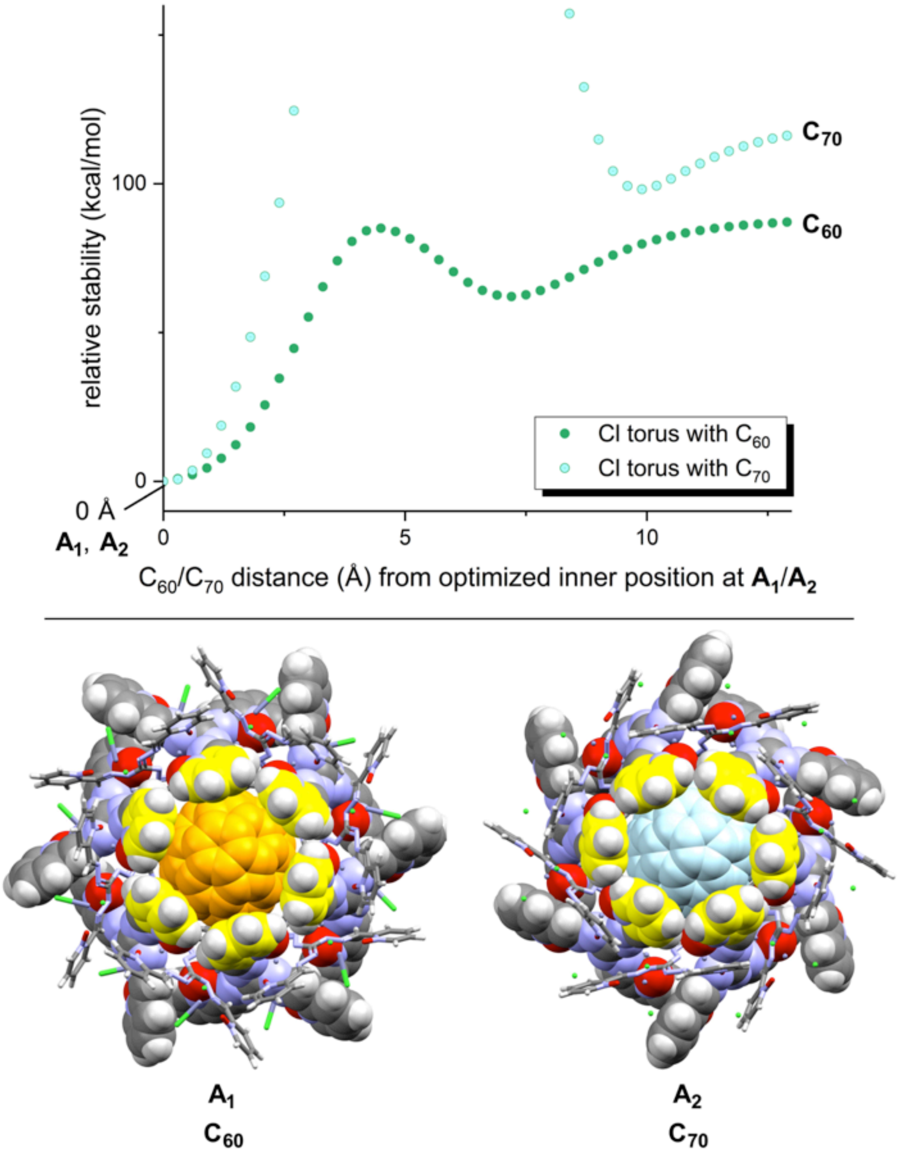

Figure 18: Top: Relative stability of a C_{60} (**A_1**, green) and C_{70} (**A_2**, blue) molecule exiting a Cl-metallacycle using stepwise increments. Bottom: The fully geometry optimized structures of each assembly. **A_1** contains C_{60} (orange) whereas **A_2** contains C_{70} (light blue). The DFT BP86/6-31G level of theory was used in gas phase with GD3BJ dispersion corrections.

For the C_{70} system there is a shallower (compared to C_{60}) local minimum around 9.9 Å (**C**) from the center optimized position of the metallacycles (Cl, Br, and NCS). A high-energy point (possibly a transition state) is found around 5.4 Å (**B**) from the center of the optimized metallacycle. This high-energy point is observed and more pronounced compared to the C_{60}-case because C_{70} is even larger and less symmetric than C_{60}, which causes a large steric repulsion upon entering the metallacycle. The metallacycle does not have the opportunity to

relax and adjust the "entry port" to the incoming C_{70} (which otherwise would have been wider) because of the nature of the single-point calculations.

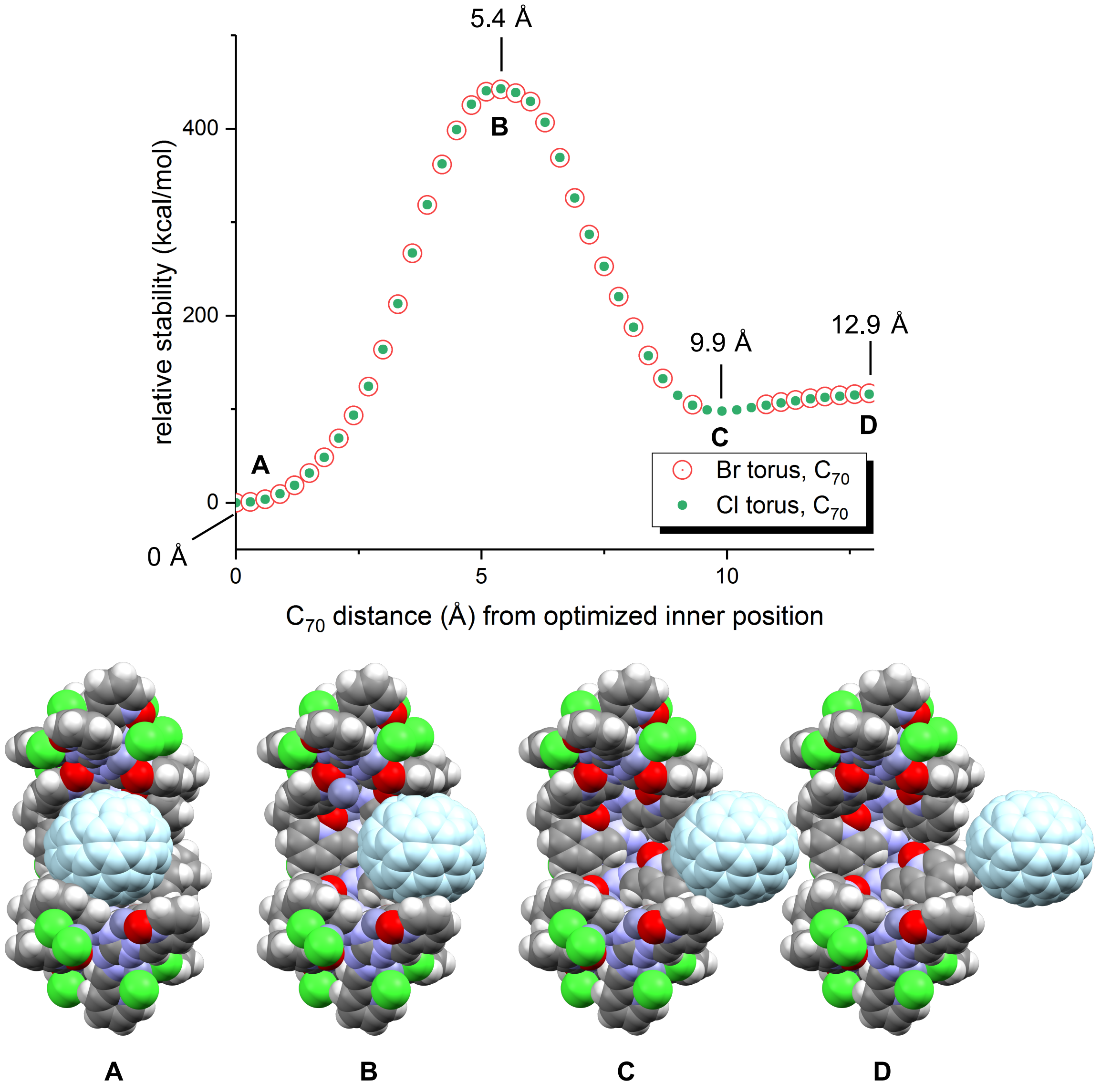

Figure 19: Relative stability of a C_{70} exiting a Cl/Br metallacycle at the BP86/6-31G/GD3BJ level of theory (gas phase). Five calculation points for the Br metallacycle did not converge.

Computational results: electrostatic potential plots & frontier orbitals

Table 1 shows the ESD and frontier orbitals of **6-8** indicating that the core of **6** and the exterior is more negative compared to both **7** and **8**. Thus, if the guest molecules are somewhat electron rich, then there should be a binding preference in the following order X = Cl >> Br > NCS, however, this ignores any effects of size and sterics. If the guest molecules are electron deficient this preference could be perturbed.

The highest occupied molecular orbitals are mainly located on the outer surface of the metallacycles but are more delocalized in character with an increasing electronegative element. For the less electronegative groups (Br and NCS) the HOMO is higher in energy and does not get close to the orbitals of the rest of the metallacycle. For **6**, the chlorides are very electronegative, bringing the occupied orbitals lower in energy and are closer to the remaining parts of the metallacycle.

Table 1. Electrostatic potential energy plots (ESD) and frontier orbitals of **6-8**.[s13]

	Cl (**6**)	Br (**7**)	NCS (**8**)
ESD	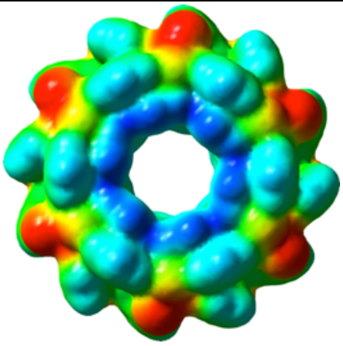	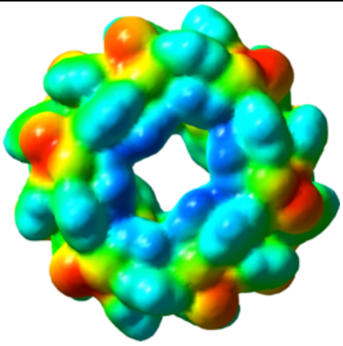	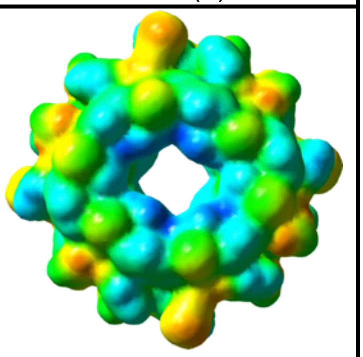
LUMO	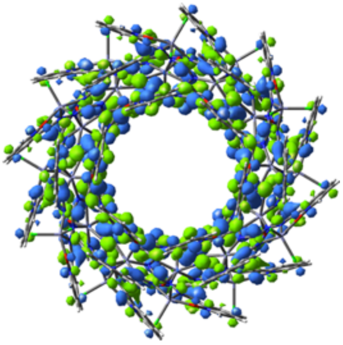	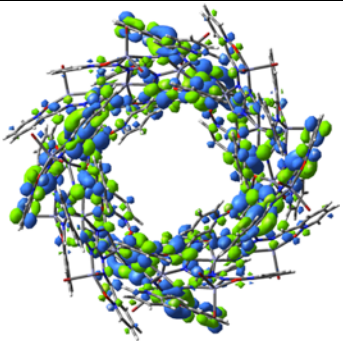	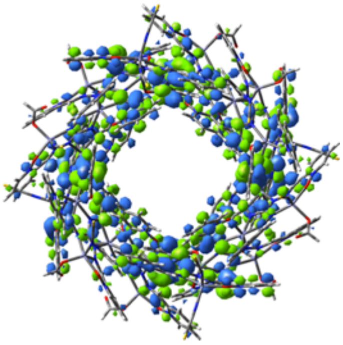
HOMO	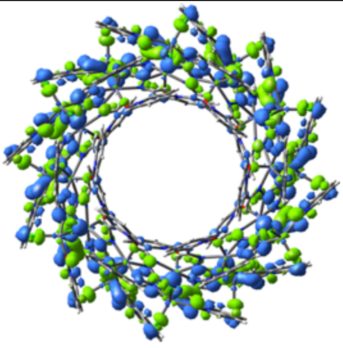	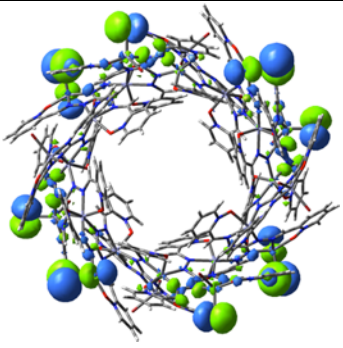	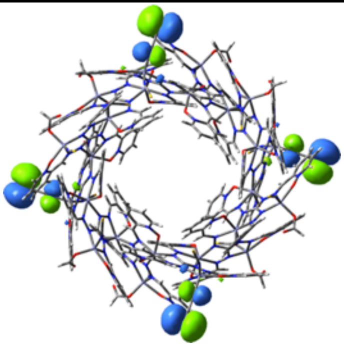

Table 2. Electrostatic potential energy plots (ESD) and frontier orbitals of three different types of filled metallacycles.

	filled Cl-metallacycle	filled Br-metallacycle	filled NCS-metallacycle
ESD	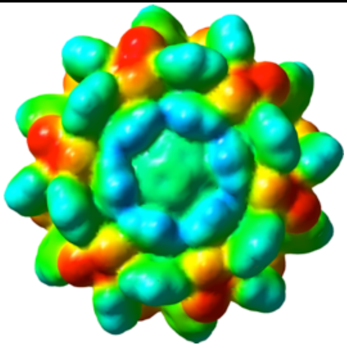	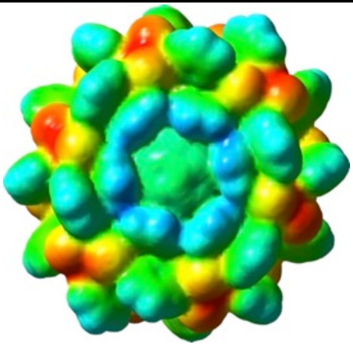	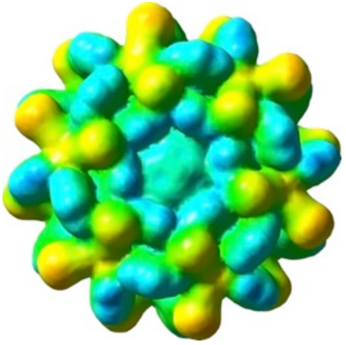
LUMO	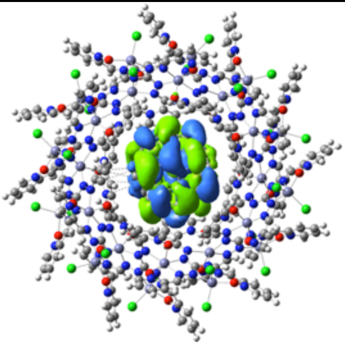	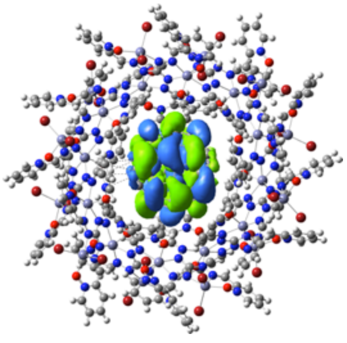	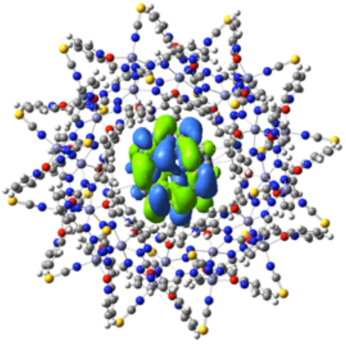
HOMO	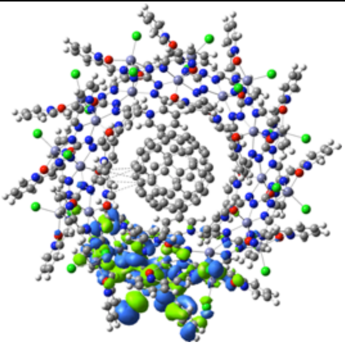	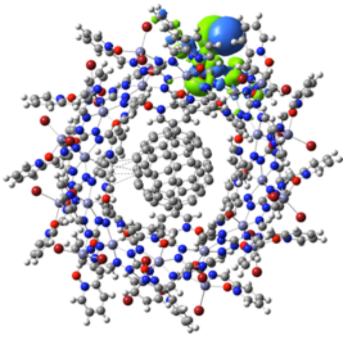	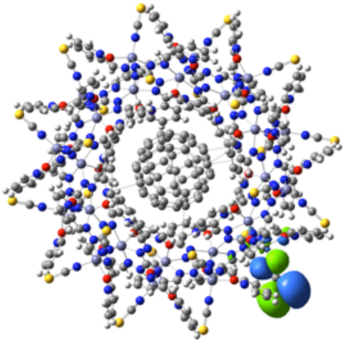

Computational results: NMR computations of **9** vs. free C_{60}, and **6**

The chemical shift of a molecule like C_{60} is time-averaged in a measured experiment, and because of its inherent symmetry, only showing *one* signal. The C_{70} analogue is less symmetric but if it too, in a free environment, tumbles allowing it to give a limited number of signals.[s14] However, in these DFT computations, only one optimized structure from *one* given conformation is computed. This results in a set of sixty ^{13}C signals being obtained, one per each carbon atom (see Table 2). All signals were therefore averaged using a normal distribution curve (Figure 22). All isotropic chemical shifts were corrected with TMS computed at the same level of theory (T_d-symmetry). Molecular structure renderings were generated with CYLView.[s18]

A variety of calculations were performed to further probe the contributions of chemical shift from the chemical environment of the metallacycle vs. structural changes in the C_{60} itself upon entering the host (Figure 21, bottom). The NMR was computed (route "**D**") for the fully geometry optimized metallacycle-C_{60} (**9**) assembly to probe the environmental impact of the metallacycle on the chemical shift of the C_{60} guest. The C_{60} geometry was then extracted and an NMR calculation were performed, however, *without* optimizing it (route "**C**"). The C_{60} molecule was then geometry optimized and subsequently had its NMR spectrum computed (route "**E**").

The average chemical shift of a free and optimized C_{60} "**E**" was computed to 144.3 ppm vs. the interacting C_{60} inside an NCS-metallacycle "**B**" (geometry from "**E**" is frozen) of 142.2 ppm. The data is shown as normal distribution curves in Figure 22, and the data is tabulated in Table 2. The average chemical shift of a free C_{60} "**C**" (geometry from "**D**" is frozen) was computed to 144.5 ppm vs. the interacting C_{60} inside an NCS-metallacycle **9** "**D**" (geometry optimized) of 142.5 ppm. The data is shown as normal distribution curves in Figure 22, and the data is tabulated in Table 2.

These data conclude that there consistently is an upfield chemical shift difference of ca. 2.0 ppm for the molecular assembly compared to the free C_{60}, no matter if it the geometry is frozen or optimized. The chemical shift difference is present *due to the chemical environment exerted by the metallacycle*. The only noticeable difference between the methods can be seen between Figure 22 and 23 in that the free C_{60}, if not optimized, has a larger spread because its geometry has been slightly distorted by being inside the C_{60} metallacycle.

Computed ^1H chemical shift to assign specific regions of the empty metallacycle (**6**) are provided in Figure 24. These computations were run on and S_{12} symmetrized metallacycle of **6** using the same methodology as prescribed above.

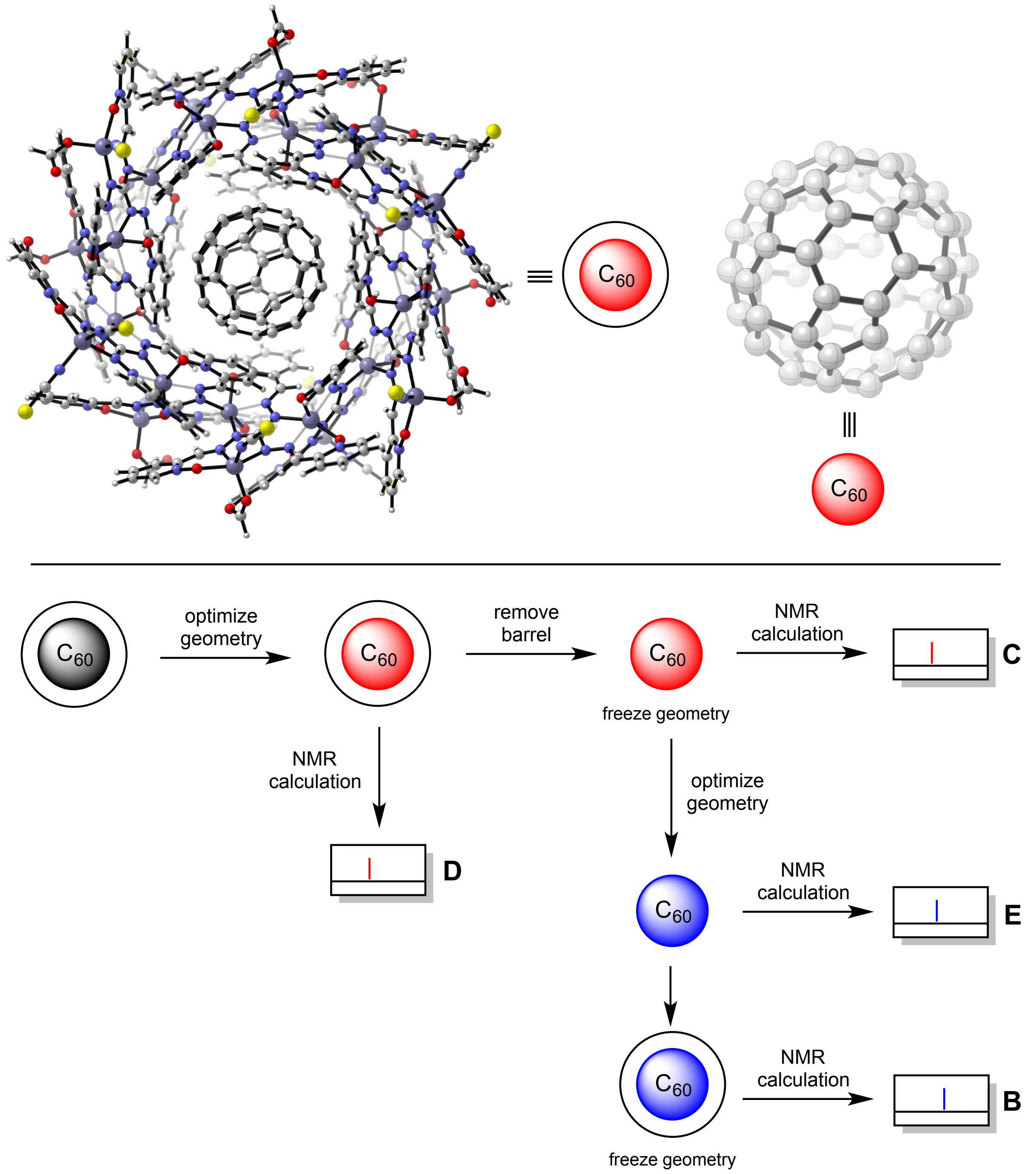

Figure 21: Model systems for ^{13}C NMR computations of free C_{60} vs. the bound C_{60} to a full Cl-metallacycle. Scheme for computing the ^{13}C chemical shift of a free vs. encapsulated C_{60}.

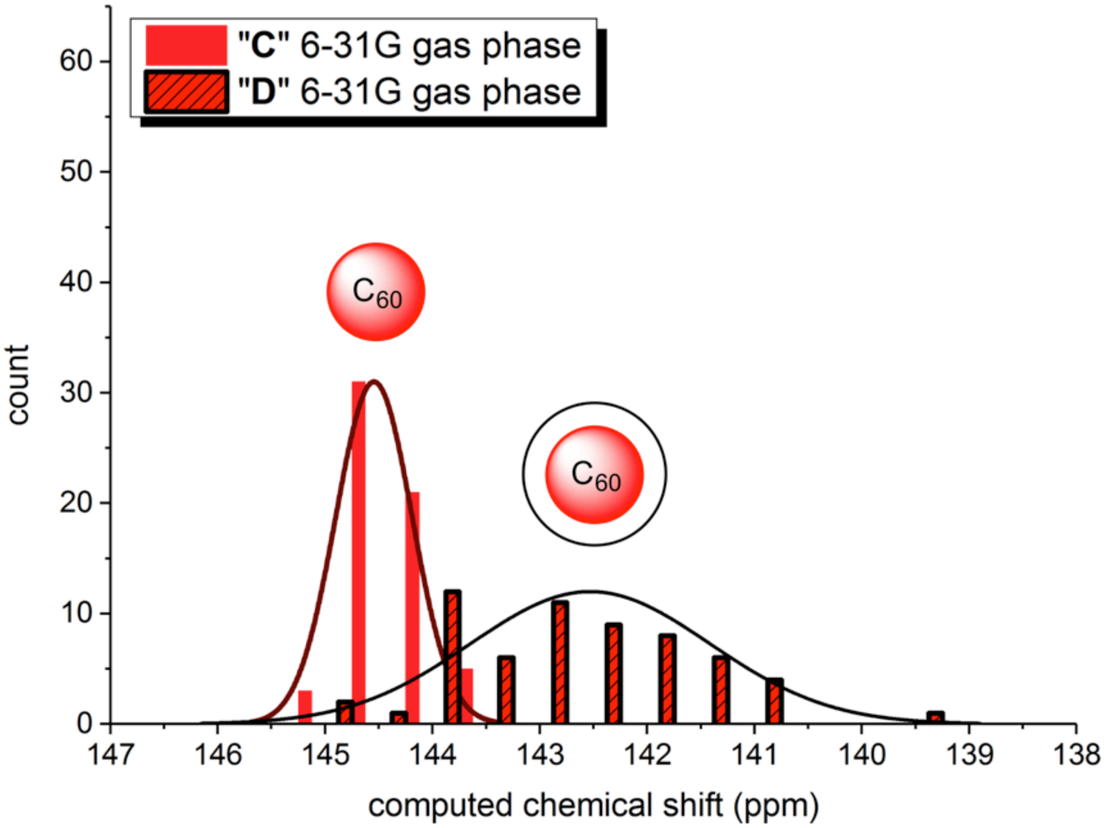

Figure 22: All computed ^{13}C isotropic chemical shifts (corrected vs. TMS) for the free C_{60} vs. the bound C_{60} to a full Cl-metallacycle using method ”**C**” and ”**D**”.

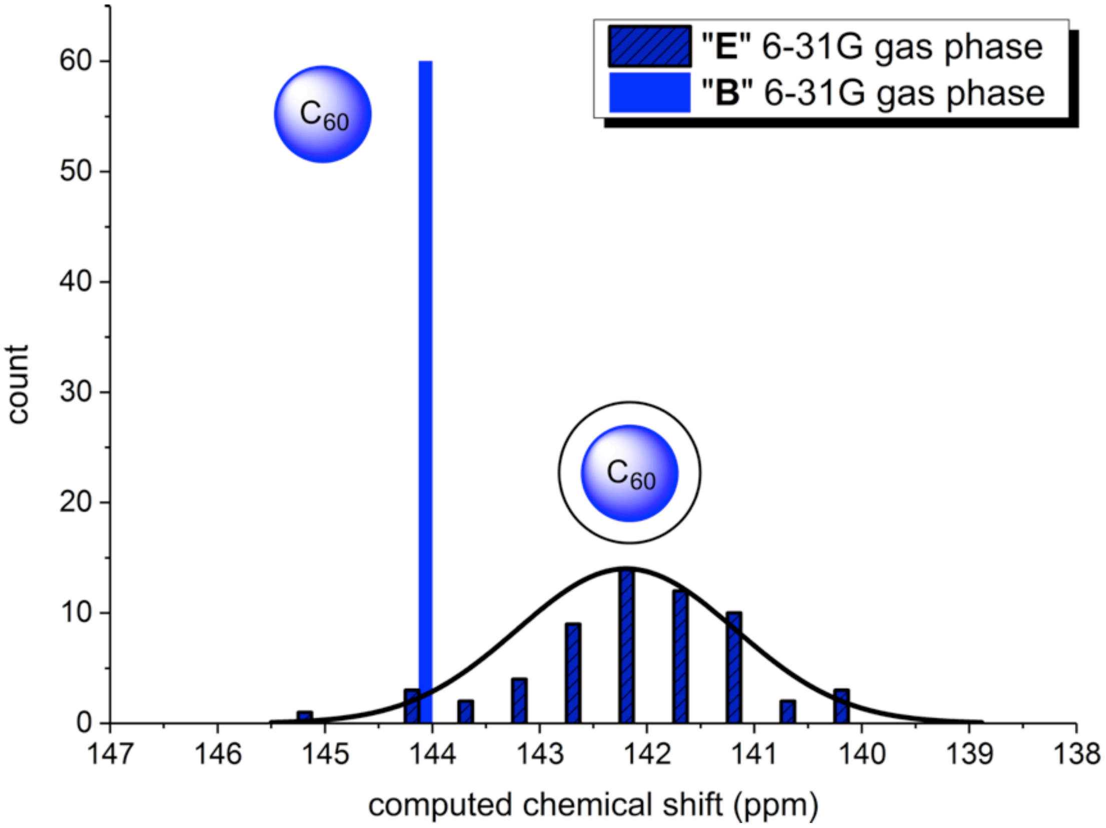

Figure 23: All computed ^{13}C isotropic chemical shifts (corrected vs. TMS) for the free C_{60} vs. the bound C_{60} to a full Cl-metallacycle using method ”**B**” and ”**E**”.

Table 2: The computed ^{13}C isotropic chemical shifts (corrected vs. TMS) for the free C_{60} vs. the interacting C_{60} inside a full NCS-metallacycle (**9**).

C_n	free C_{60} (δ,ppm)	free C_{60} (δ,ppm)	**9** (δ,ppm)	**9** (δ,ppm)
	route "**C**"	route "**E**"	route "**B**"	route "**D**"
1	144.3420	144.2892	141.7064	142.0247
2	144.7371	144.2648	141.9275	142.0085
3	144.7460	144.2368	141.6247	141.8556
4	144.8901	144.2432	140.4105	141.6301
5	145.1326	144.2731	143.9110	143.7152
6	144.3687	144.2662	142.9578	141.2507
7	143.6585	144.2726	142.6993	139.3181
8	144.6746	144.2875	142.9410	142.3371
9	144.6629	144.2396	141.0128	141.1945
10	144.9588	144.2825	142.0370	143.5983
11	144.3591	144.2660	141.9561	141.7817
12	144.7210	144.2776	141.1380	142.9385
13	144.7135	144.2485	142.3051	143.8634
14	144.8330	144.2847	141.9738	143.1519
15	144.7611	144.2153	144.2108	142.8083
16	144.9438	144.2675	142.7500	142.9108
17	144.6630	144.2897	143.5271	142.5358
18	144.2630	144.2655	142.3808	142.4104
19	144.0755	144.3154	142.5896	143.2678
20	144.8360	144.2807	142.0199	143.4189
21	144.6558	144.2759	141.3855	144.6137
22	144.4341	144.2667	145.0686	142.7433
23	144.7956	144.2633	141.2011	142.1662
24	145.2738	144.2656	142.1634	142.7954
25	144.9050	144.2602	140.7605	144.0600
26	144.1388	144.2534	142.0726	143.7703
27	144.4181	144.2939	142.3702	143.9649
28	143.6035	144.2782	142.3518	141.6464
29	143.9687	144.2752	140.4779	141.0374
30	144.8146	144.2915	143.3435	143.1727
31	144.6101	144.2737	141.6451	142.7881
32	145.1799	144.2734	142.4212	142.8953
33	144.7045	144.2626	142.5087	143.5937
34	144.8348	144.2859	142.4898	142.5208
35	144.5545	144.2605	141.2248	141.9117
36	144.3113	144.2859	141.7602	141.6000
37	144.2418	144.2550	144.4688	140.7042
38	144.3761	144.3134	142.4862	143.7684
39	144.5389	144.2734	142.1076	144.6340
40	144.3078	144.2757	143.4072	142.7655
41	144.3477	144.2646	141.8927	142.4076
42	144.7890	144.2878	141.1916	143.6226
43	144.9148	144.2726	141.6198	142.4538
44	144.3075	144.2497	144.3317	143.9729
45	144.5569	144.2665	143.3221	143.8489
46	144.3507	144.2533	142.8359	140.7842
47	144.2924	144.2821	140.4769	142.0997
48	144.4967	144.2294	141.5584	141.7373
49	144.2762	144.2595	141.3669	140.8596
50	144.9184	144.2839	141.6500	142.4846
51	144.7676	144.2511	141.2918	141.2482
52	144.8936	144.2822	141.0693	143.7518
53	144.3458	144.2539	140.7056	140.7083
54	144.2295	144.2686	142.3285	143.3722
55	143.7514	144.2576	142.6900	141.5540
56	144.7443	144.3071	143.2348	142.8379
57	144.7343	144.2423	142.7701	143.0527
58	143.8601	144.2962	141.9923	143.5120
59	144.4497	144.2650	141.144	141.3082
60	144.6636	144.2645	142.2497	141.0024

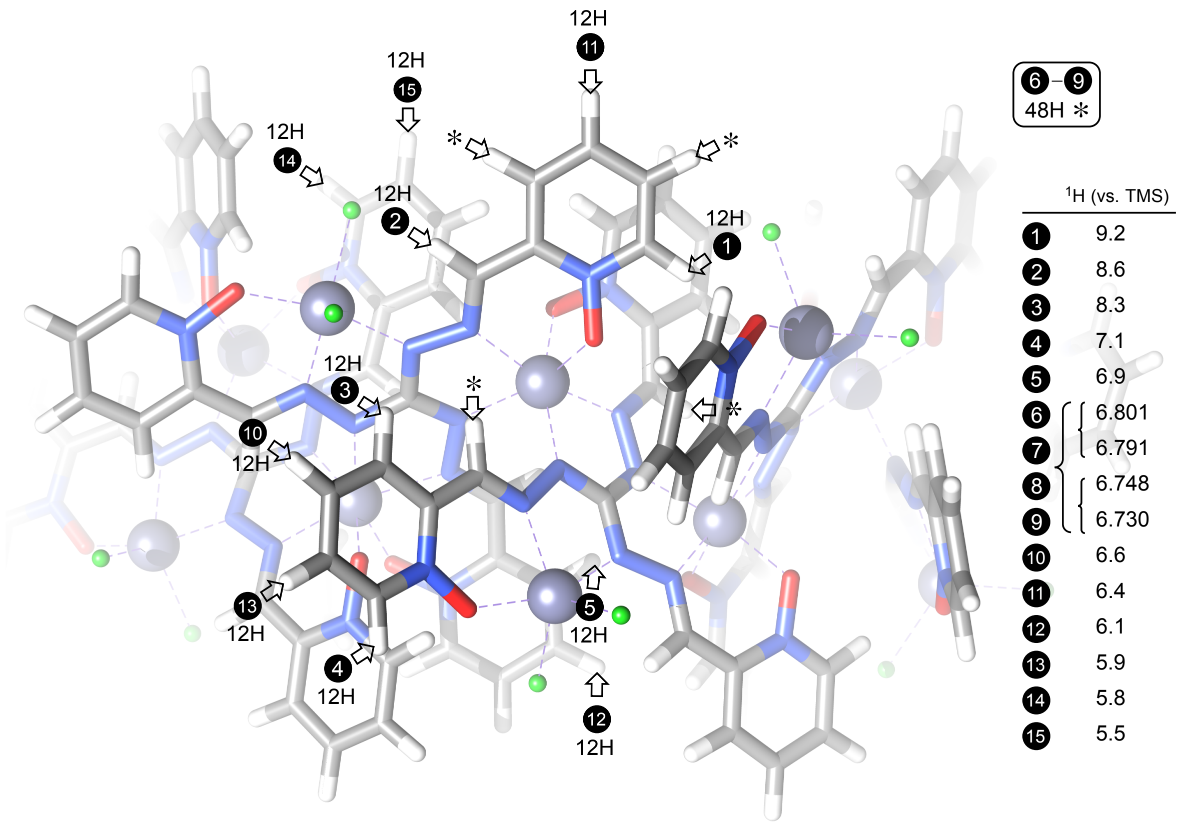

Figure 24. Computed ^{1}H chemical shifts of a S_{12} symmetrized chloride metallacycle (**6**).

Computational results: IR of **6**

It is necessary to perform a frequency calculation in order to compute an IR-spectrum. For most of the studied compounds (and all filled metallacycles) this was not possible as explained on p. 17. However, one of the C_2-symmetrized structures of **6** converged and showed only positive frequencies, indicating that the structure is a local minimum.

The computed spectrum (in gas phase) was overlaid with experimental data of **6** (Figure 25). The spectrum shows similar fingerprint absorptions with most peaks completely reproduced in the computed spectrum in the 500-1500 cm^{-1} region. Even the high intensity peaks at 1440 cm^{-1} are obtained with a similar shoulder pattern near 1300 cm^{-1}. The computed peak at 3100 cm^{-1} is buried in the absorption in the experimental spectra but still slightly visible despite H-bonded broad absorptions. Most of the peaks, including the high-intensity peak at 1440 cm^{-1}, are complex C-H absorptions from the aromatic pyridinyl groups.

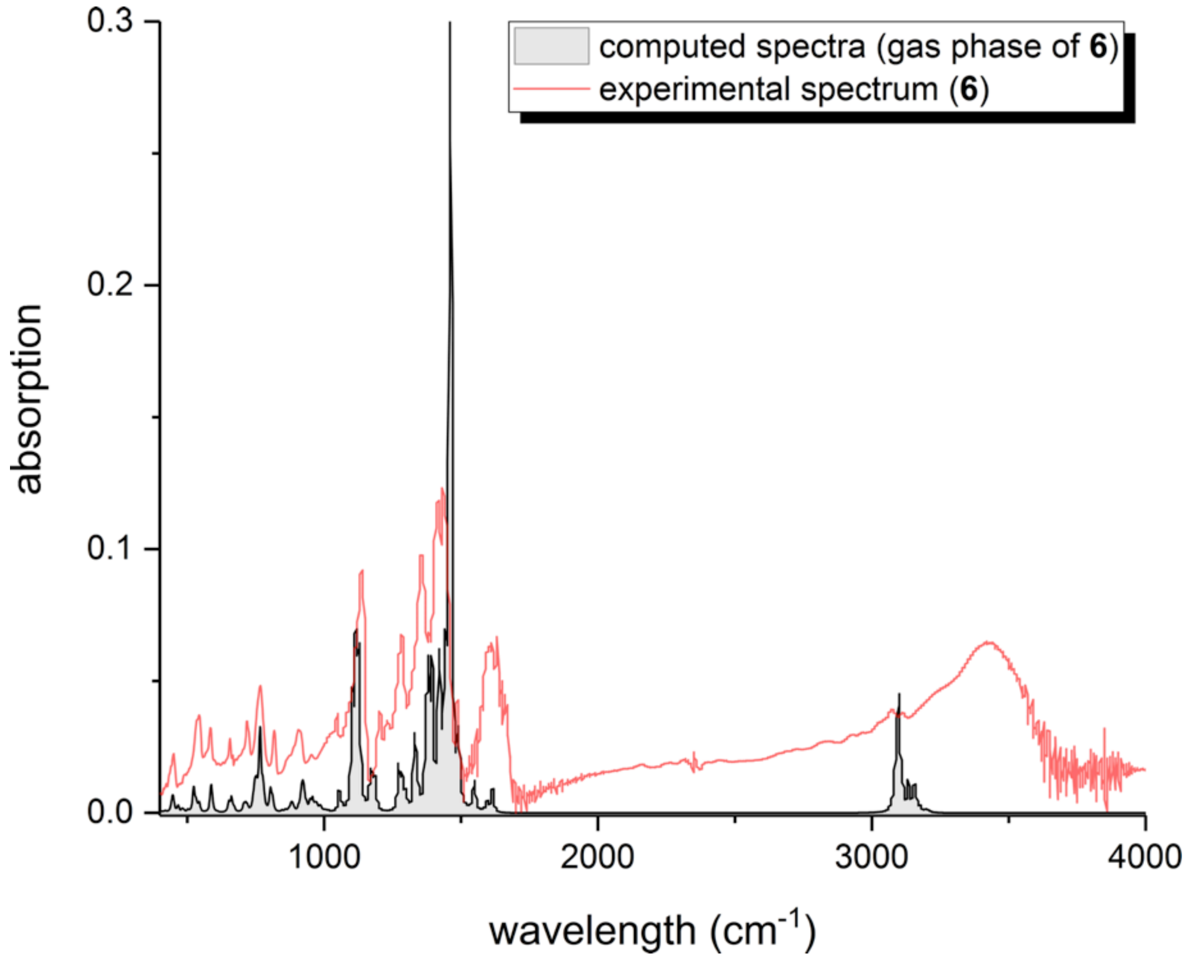

Figure 25: A computed IR spectrum (black) overlaid with an experimental spectrum (red) of **6**.

Literature

[s1] Gaussian 16, *Revision B.01*, Frisch, M. J.; Trucks, G. W.; Schlegel, H. B.; Scuseria, G. E.; Robb, M. A.; Cheeseman, J. R.; Scalmani, G.; Barone, V.; Petersson, G. A.; Nakatsuji, H.; Li, X.; Caricato, M.; Marenich, A. V.; Bloino, J.; Janesko, B. G.; Gomperts, R.; Mennucci, B.; Hratchian, H. P.; Ortiz, J. V.; Izmaylov, A. F.; Sonnenberg, J. L.; Williams-Young, D.; Ding, F.; Lipparini, F.; Egidi, F.; Goings, J.; Peng, B.; Petrone, A.; Henderson, T.; Ranasinghe, D.; Zakrzewski, V. G.; Gao, J.; Rega, N.; Zheng, G.; Liang, W.; Hada, M.; Ehara, M.; Toyota, K.; Fukuda, R.; Hasegawa, J.; Ishida, M.; Nakajima, T.; Honda, Y.; Kitao, O.; Nakai, H.; Vreven, T.; Throssell, K.; Montgomery, J. A., Jr.; Peralta, J. E.; Ogliaro, F.; Bearpark, M. J.; Heyd, J. J.; Brothers, E. N.; Kudin, K. N.; Staroverov, V. N.; Keith, T. A.; Kobayashi, R.; Normand, J.; Raghavachari, K.; Rendell, A. P.; Burant, J. C.; Iyengar, S. S.; Tomasi, J.; Cossi, M.; Millam, J. M.; Klene, M.; Adamo, C.; Cammi, R.; Ochterski, J. W.; Martin, R. L.; Morokuma, K.; Farkas, O.; Foresman, J. B.; Fox, D. J. Gaussian, Inc., Wallingford CT, **2016**.

[s2] Texas A&M University High Performance Research Computing Center using the "TERRA" supercomputer with 256 28-core compute nodes, each with 64GB RAM, and running on the Linux (CentOS 7) on a General Parallel File System (GPFS).

[s3] (a) "Density-functional exchange-energy approximation with correct asymptotic behavior", Becke, A. D. *Phys. Rev. A*, **1988**, *38*, 3098-3100. (b) "Density-functional approximation for the correlation energy of the inhomogeneous electron gas", Perdew, J. P. *Phys. Rev. B*, **1986**, *33*, 8822-8824.

[s4] (a) "Density-functional thermochemistry. III. The role of exact exchange", Becke, A. D. J. Chem. *Phys.* **1993**, 98, 5648-5652. (b) "Development of the Colle-Salvetti correlation energy formula into a functional of the electron density", Lee, C.; Yang, W.; Parr, R. G. *Phys. Rev. B*, **1988**, *37*, 785-789.

[s5] Pople basis set reference for "6-31G". An addition of "*d*" adds polarization functions of *d*-type to "heavy" atoms like C, N and O whereas "*p*" adds *p*-type polarization functions to C, N, O, and H atoms. "+/++" indicates added diffuse functions: (a) "Self-Consistent Molecular-Orbital Methods. IX. An Extended Gaussian-Type Basis for Molecular-Orbital Studies of Organic Molecules", Ditchfield, R.; Hehre, W. J.; Pople, J. A. *J. Chem. Phys.* **1971**, *54*, 724-728. (b) "Self-Consistent Molecular Orbital Methods. XII. Further Extensions of Gaussian-Type Basis Sets for Use in Molecular Orbital Studies of Organic Molecules", Hehre, W. J.; Ditchfield, R.; Pople, J. A. *J. Chem. Phys.* **1972**, *56*, 2257-2261. (c), "The Influence of Polarization Functions on Molecular Orbital Hydrogenation Energies", Hariharan, P. C.; Pople, J. A. *Theor. Chem. Acc.* **1973**, *28*, 213-222. (d) "Accuracy of AH_n equilibrium geometries by single determinant molecular orbital theory", Hariharan, P. C.; Pople, J. A. *Mol. Phys.* **1974**, *27*, 209-214. (e) "The Isomers of Silacyclopropane", Gordon, M. S. *Chem. Phys. Lett.* **1980**, *76*, 163-168. (f) "Self-consistent molecular orbital methods. XXIII. A

polarization-type basis set for second-row elements", Francl, M. M.; Pietro, W. J.; Hehre, W. J.; Binkley, J. S.; Gordon, M. S.; DeFrees, D. J.; Pople, J. A. *J. Chem*. Phys. **1982**, *77*, 3654-3665. (g) "Compact Contracted Basis Sets for Third-Row Atoms: Ga-Kr", Binning, Jr. R. C.; Curtiss, L. A. *J. Comp. Chem*. **1990**, *11*, 1206-1216. (h) "Extension of Gaussian-2 (G2) theory to molecules containing third-row atoms K and Ca", Blaudeau, J.-P.; McGrath, M. P.; Curtiss, L. A.; Radom, L. *J. Chem. Phys.* **1997**, *107*, 5016-5021. (i) "6-31G* basis set for atoms K through Zn", Rassolov, V. A.; Pople, J. A.; Ratner, M. A.; Windus, T. L. *J. Chem. Phys*. **1998**, *109*, 1223-1229. (j) "6-31G* Basis Set for Third-Row Atoms", Rassolov, V. A.; Ratner, M. A.; Pople, J. A.; Redfern, P. C.; Curtiss, L. A. *J. Comp. Chem*. **2001**, *22*, 976-984.

[s6] Pople basis set reference for "6-311G". An addition of "*d*" adds polarization functions of *d*-type to heavy atoms, "*p*" adds *p*-type polarization functions to heavy atoms *and* H atoms. "+/++" indicates added diffuse functions: (a) "Contracted Gaussian basis sets for molecular calculations. I. Second row atoms, *Z* = 11-18", McLean, A. D.; Chandler, G. S.; *J. Chem. Phys*. **1980**, *72*, 5639-5648. (b) "Self-consistent molecular orbital methods. XX. A basis set for correlated wave functions", Raghavachari, K.; Binkley, J. S.; Seeger, R.; Pople, J. A. *J. Chem. Phys*. **1980**, *72*, 650-654.

[s7] "Effect of the damping function in dispersion corrected density functional theory", Grimme, S.; Ehrlich, S.; Goerigk, L. *J. Comp. Chem*. **2011**, *32*, 1456-1465.

[s8] "Universal solvation model based on solute electron density and a continuum model of the solvent defined by the bulk dielectric constant and atomic surface tensions", Marenich, A. V.; Cramer, C. J.; Truhlar, D. G. *J. Phys. Chem. B*, **2009**, *113*, 6378-6396.

[s9] (a) "Fullerenes in Liquid Media: An Unsettling Intrusion into the Solution Chemistry", Mchedlov-Petrossyan, N. O. *Chem. Rev*. **2013**, *113*, 5149-5193. (b) "Accurate Intermolecular Potential for the C_{60} Dimer: The Performance of Different Levels of Quantum Theory", Sharapa, D. I.; Margraf, J. T.; Hesselmann, A.; Clark, T. *J. Chem. Theory Comput*. **2017**, *13*, 274-285.

[s10] "Semiempirical GGA-type density functional constructed with a long-range dispersion correction", Grimme, S. *J. Comp. Chem*. **2006**, *27*, 1787-1799.

[s11] "A consistent and accurate *ab initio* parameterization of density functional dispersion correction (DFT-D) for the 94 elements H-Pu", Grimme, S.; Antony, J.; Ehrlich, S.; Krieg, H. *J. Chem. Phys*. **2010**, *132*, 154104.

[s12] "Mercury CSD 2.0 – new features for the visualization and investigation of crystal structures", Macrae, C. F.; Bruno, I. J.; Chisholm, J. A.; Edgington, P. R.; McCabe, P.; Pidcock, E.; Rodriguez-Monge, L.; Taylor, R.; van de Streek, J.; Wood, P. A. *J. Appl. Crystallogr*. **2008**, *41*, 466-470.

[s13] *GaussView, Version 6*, Dennington, R.; Keith, T. A.; Millam, J. M. Semichem Inc., Shawnee Mission, KS, **2016**.

[s14] (a) "On accuracy of the ^{13}C NMR chemical shift GIAO calculations of fullerene C_{60} derivatives at PBE/3ζ approach", Tulyabaev, A. R.; Khalilov, L. M. *Comput. Theor. Chem*.

2011, *976*, 12-18. (b) "Density functional theory on ^{13}C NMR chemical shifts of fullerene", P. Christy, A.; Peter A. J.; Lee, C. W. *Solid State Commun*. **2018**, *283*, 22-26. (c) "Theoretical ^{13}C NMR Spectra of IPR Isomers of Fullerenes C_{60}, C_{70}, C_{72}, C_{74}, C_{76}, and C_{78} Studied by Density Functional Theory", Sun, G.; Kertesz. M. *J. Phys. Chem. A*, **2000**, *104*, 7398-7403. (d) "Endohedral ^{1}H NMR Chemical Shifts of H_2-, H_2O- and NH_3-Encapsulated Fullerene Compounds: Accurate Calculation and Prediction", Guan-Wu Wang, G.-W.; Wu, P.; Tian, Z.-G. *Eur. J. Org. Chem*. **2009**, 1032-1041. (e) "Theoretical Prediction of the Host-Guest Interactions Between Novel Photoresponsive Nanorings and C_{60}: A Strategy for Facile Encapsulation and Release of Fullerene", Yuan, K.; Dang, J.-S.; Guo, Y.-J.; Zhao, X. *J. Comput. Chem*, **2015**, *36*, 518-528.

[s15] "Computational Prediction of ^{1}H and ^{13}C Chemical Shifts: A Useful Tool for Natural Product, Mechanistic, and Synthetic Organic Chemistry", Lodewyk, M. W.; Siebert, M. R.; Tantillo, D. J. *Chem. Rev*. **2012**, *112*, 1839-1862.

[s16] "Evaluation of the Factors Impacting the Accuracy of ^{13}C NMR Chemical Shift Predictions using Density Functional Theory – The Advantage of Long-Range Corrected Functionals", Iron, M. A. *J. Chem. Theory Comput*. **2017**, *13*, 5798-5819.

[s17] (a) "The quantic theory of inter-atomic currents in aromatic combinations", F. London, F. *J. Phys. Radium*, **1937**, *8*, 397-409. (b) "Perturbation Theory for Fock-Dirac Density Matrix", McWeeny, R. *Phys. Rev*. **1962**, *126*, 1028. (c) "Self-consistent perturbation theory of diamagnetism. 1. Gauge-invariant LCAO method for NMR chemical shifts", Ditchfield, R. *Mol. Phys*. **1974**, *27*, 789-807. (d) "Efficient Implementation of the Gauge-Independent Atomic Orbital Method for NMR Chemical Shift Calculations", Wolinski, K.; Hilton, J. F.; Pulay, P. *J. Am. Chem. Soc*. **1990**, *112*, 8251-8260. (e) "A Comparison of Models for Calculating Nuclear Magnetic Resonance Shielding Tensors", Cheeseman, J. R.; Trucks, G. W.; Keith, T. A.; Frisch, M. J. *J. Chem. Phys*. **1996**, *104*, 5497-5509.

[s18] "CYLview, 1.0b"; Legault, C. Y., Université de Sherbrooke, **2009** (http://www.cylview.org)

[s19] "An idealized molecular geometry library for refinement of poorly behaved molecular fragments with constraints", I. A. Guzei, *J. Appl. Cryst.* **2014**. *47*, 806-809.

[s20] "Local curvature and stability of two-dimensional systems", J. Guan, Z. Jin, Z. Zhu, C. Chuang, B.-Y. Jin, D. Tománek, *Phys. Rev. B* **2014**, *90*, 245403.
